# Temporal dynamics of total and active root-associated diazotrophic communities in field-grown rice

**DOI:** 10.3389/fmicb.2022.1016547

**Published:** 2022-10-13

**Authors:** Xue Luo, Xianfeng Ye, Wenhui Wang, Yang Chen, Zhoukun Li, Yanxin Wang, Yan Huang, Wei Ran, Hui Cao, Zhongli Cui

**Affiliations:** ^1^Key Laboratory of Agricultural Environmental Microbiology, Ministry of Agriculture and Rural Affairs, College of Life Science, Nanjing Agricultural University, Nanjing, China; ^2^School of Life Sciences, Anhui Agricultural University, Hefei, China; ^3^Key Laboratory of Microbial Resources Collection and Preservation, Ministry of Agriculture and Rural Affairs, Beijing, China; ^4^Jiangsu Collaborative Innovation Center for Solid Organic Waste Utilization, College of Resources and Environmental Sciences, Nanjing Agricultural University, Nanjing, China

**Keywords:** diazotrophs, rhizosphere soil, rice root, community succession, transcription level

## Abstract

Plant-associated nitrogen-fixing microorganisms (diazotrophs) are essential to host nutrient acquisition, productivity and health, but how host growth affects the succession characteristics of crop diazotrophic communities is still poorly understood. Here, Illumina sequencing of DNA- and RNA-derived *nifH* genes was employed to investigate the dynamics of total and active diazotrophic communities across rhizosphere soil and rice roots under four fertilization regimes during three growth periods (tillering, heading and mature stages) of rice in 2015 and 2016. Our results indicated that 71.9–77.2% of the operational taxonomic units (OTUs) were both detected at the DNA and RNA levels. According to the nonmetric multidimensional scaling ordinations of Bray–Curtis distances, the variations in community composition of active rhizosphere diazotrophs were greater than those of total rhizosphere diazotrophs. The community composition (*β*-diversity) of total and active root-associated diazotrophs was shaped predominantly by microhabitat (niche; *R*^2^ ≥ 0.959, *p* < 0.001), followed by growth period (*R*^2^ ≥ 0.15, *p* < 0.001). The growth period had a stronger effect on endophytic diazotrophs than on rhizosphere diazotrophs. From the tillering stage to the heading stage, the *α*-diversity indices (Chao1, Shannon and phylogenetic diversity) and network topological parameters (edge numbers, average clustering coefficient and average degree values) of total endophytic diazotrophic communities increased. The proportions of OTUs shared by the total rhizosphere and endophytic diazotrophs in rhizosphere diazotrophs gradually increased during rice growth. Moreover, total diazotrophic *α*-diversity and network complexity decreased from rhizosphere soil to roots. Collectively, compared with total diazotrophic communities, active diazotrophic communities were better indicators of biological response to environmental changes. The host microhabitat profoundly drove the temporal dynamics of total and active root-associated diazotrophic communities, followed by the plant growth period.

## Importance

Diazotrophs in the rhizosphere soil and roots of gramineous plants can not only provide nitrogen for host plants through biological nitrogen fixation but be used as potential biological control agents, biological regulators and biodegradation agents. However, the succession patterns of rice rhizosphere and root endophytic diazotrophic communities are still poorly understood. Illumina sequencing results of DNA- and RNA-derived *nifH* genes revealed that for either total or active diazotrophs, the growth period has a greater effect on the diazotrophic composition than the fertilization regime, and the *α*-diversity, community composition and co-occurrence networks of rhizosphere and endophytic diazotrophs show different succession patterns. Our study provides novel insights into the temporal dynamics of root-associated diazotrophic diversity and co-occurrence networks in field-grown rice.

## Introduction

As a critical macronutrient, nitrogen (N) often limits crop yields in agricultural ecosystems (Pankievicz et al., 2019). Over the past several decades, while chemical fertilizer addition has increased crop production, fertilizer overuse has caused serious environmental damage (Gong et al., 2011; Shahzad et al., 2019). Biological nitrogen fixation (BNF) is a process of reducing atmospheric dinitrogen to biologically available ammonium by nitrogen-fixing bacteria and archaea. BNF with high nitrogen-use efficiency (NUE) is an available and eco-friendly way to provide N for plants (Zhang X. et al., 2015). Previous studies have demonstrated that diazotrophs contribute to crop fitness and productivity through a variety of mechanisms, including enhancing plant nutrient absorption (Baldani et al., 2000), producing plant hormones (Shabanamol et al., 2018) and antimicrobials (Weselowski et al., 2016) and improving abiotic stress tolerance (Naveed et al., 2014). Compared with the increasing knowledge of diazotrophic community composition and metabolic capacity, much less is known about temporal dynamics of diazotrophic communities. Moreover, one of the prerequisites for harnessing plant-associated diazotrophs to maximize crop production is to illustrate their formation and succession processes.

Niche differentiation of microbiome at the rhizosphere soil–root interface has been a hotspot in the study of plant–microbe interactions (Beckers et al., 2017; Shi et al., 2021). For instance, great differences in the rhizosphere, rhizoplane and root endophytic bacterial communities of rice were found, and the *α*-diversity gradually decreased from the rhizosphere to the rhizoplane and then to the endosphere (Edwards et al., 2015). Root-associated diazotrophs include diazotrophs that colonize the rhizosphere soil (rhizosphere diazotrophs), the rhizoplane (rhizoplane diazotrophs) and the roots (endophytic diazotrophs; Bergholz et al., 2001; Liu et al., 2020). Previous studies have shown that there are large differences in the *nifH* gene abundance and diazotrophic community between rhizosphere soil and roots of rice (Wartiainen et al., 2008), maize (Berge et al., 1991), wheat (Rilling et al., 2018) and mangrove (Liu et al., 2020) ecosystems. In contrast to the knowledge concerning the microhabitat differentiation of the rhizosphere and endophytic microbiome, a robust understanding of the community composition of the rhizosphere and endophytic diazotrophs in field conditions has remained elusive.

In addition to microhabitat, many biotic and abiotic factors affect the diversity of diazotrophs in agricultural ecosystems, such as growth period (Wang J. et al., 2016), plant genotype (Zhang Y. et al., 2017), fertilization practice (Wang et al., 2017; Lin et al., 2018) and edaphic conditions (Jia et al., 2020). Previous studies revealed that the sampling time played a larger role in influencing the diazotrophic community structures than the host species, planting density and fertilization practice (Wang J. et al., 2016; Xiao et al., 2020). Shifts in diazotrophic community composition with growth period (sampling time) are often correlated with temperature, precipitation and plant developmental conditions, which directly or indirectly regulate the structures of the diazotrophic communities (Orr et al., 2012; Yeager et al., 2012). However, most of the related studies only considered one or a few factors. Thus, we are working on a systematic understanding of how microhabitat, growth period and fertilization regime interactively affect the community composition and co-occurrence networks of root-associated diazotrophs. In addition, the establishment of the rhizosphere and endophytic microbiota is a dynamic process that plants participate in and regulate (Bulgarelli et al., 2013). Previous studies have shown that rice root-associated microbiota dramatically vary at the vegetative stage and stabilize at the reproductive stage (Edwards et al., 2018; Zhang et al., 2018). By using denaturing gradient gel electrophoresis (DGGE) of amplified *nifH* genes, Monteiro et al. (2011) found that the community composition of rhizosphere diazotrophs varied with the early root growth of vetiver and tended to be stable after 3 months of growth. However, we still know little about the succession processes of rhizosphere and endophytic diazotrophs in paddy fields during rice cultivation.

Certain studies have characterized root-associated diazotrophs using the high-throughput sequencing technique of the DNA-derived *nifH* gene under different environmental conditions (Shiozaki et al., 2018; Zhou et al., 2021). However, microbes are highly dormant in the environment, and the taxa detected in the DNA libraries include DNA from living or dormant cells as well as extracellular DNA from lysed or degraded cells (Chen et al., 2016; Nagler et al., 2018). Therefore, DNA-derived sequencing analysis can lead to inaccurate interpretation of the sequencing data in the ecological researches (Nawaz et al., 2019). On the contrary, RNA-derived sequencing analysis targets the metabolically active part of the total microflora and would provide more accurate information about the functional microbes (Li et al., 2019). A few studies have shown that the abundance, diversity and community composition of RNA-derived microbial communities were different from those of DNA-derived microbial communities (Baldrian et al., 2012; Cardoso et al., 2017; Li et al., 2017; Yang et al., 2019). De Vrieze et al. (2018) found that the active microbial community more accurately reflected the anaerobic digestion process than the total microbial community. At present, our knowledge concerning active diazotrophic communities remains very limited in paddy rice systems (Wartiainen et al., 2008; Tang et al., 2017). Thus, it is worthwhile to investigate the community of rice root-associated diazotrophs at the transcript level.

Herein, we aimed to compare the diversity and co-occurrence networks of the active (RNA-derived) and total (DNA-derived) diazotrophs and assess how microhabitat, growth period and fertilization regime interactively shape the community composition and succession of rice root-associated diazotrophs. We hypothesized that (1) compared with DNA-derived *nifH* gene sequencing, RNA-derived *nifH* gene sequencing more accurately elucidates diazotrophic communities; (2) the growth period has a greater effect on the diazotrophic composition than the fertilization regime, and (3) the community composition and co-occurrence networks of rhizosphere and endophytic diazotrophs might show different succession patterns.

## Materials and methods

### Field experimental design

The field experiment was located in Jintan city, Jiangsu Province, China (31°39′N, 119°28′E). The station is located in an area with an annual average temperature of 15.3°C and a mean annual precipitation of 1063.6 mm. The soil type was classified as typical Clay loamy Fe-leachic-gleyic-stagnic anthrosol, with a long-term annual rotation of winter wheat (*Triticum aestivum* L.) and summer rice (*Oryza sativa* L.; Liu et al., 2015). The fertilization experiment started in 2010 using a randomized block design of four replicates of seventeen fertilization regimes as previously described (Zhao et al., 2014; Supplementary Figure S1A). Cement ridges 30 cm wide and 100 cm deep helped keep blocks separate from one another. Each plot was 40 m^2^ in area (5 m × 8 m). We chose four fertilization regimes: no fertilization (CK), 100% chemical fertilizers (NPK), 50% chemical fertilizers plus 6,000 kg/ha pig manure (NPKM) and 100% chemical fertilizers plus 8,000 kg/ha crop straw (NPKS). The 100% chemical fertilizer treatment included N (300 kg/ha), P_2_O_5_ (120 kg/ha) and K_2_O (100 kg/ha). In the NPKM treatment, pig manure contained 138 kg/ha N, 78 kg/ha P, 60 kg/ha K, 2724 kg/ha organic matter with moisture content of 29.1% (Liu et al., 2015). In the NPKS treatment, rice straw contained 57.2 kg/ha N, 12.1 kg/ha P, 117.7 kg/ha K, 9086 kg/ha organic matter with moisture content of 30.7%. All P, K, manure fertilizers and crop straw were applied as basal fertilizers before planting, whereas N fertilizers were used as basal fertilizers and supplementary fertilizers (basal fertilizers: tillering supplementary fertilizers: panicle supplementary fertilizers = 4:3:3).

### Sample collection

As shown in Supplementary Figure S1B, in 2015, sample collections were performed on 8 July, 1 September and 17 October, corresponding to the rice tillering stage, heading stage and mature stage. Block I, block II and block III were chosen for each time point, and for each fertilization regime, 2 rice plants were randomly collected from the sampling region of each replicate plot. Each plant was a biological replicate. Thus, there were 6 biological replicates per fertilization regime in 2015. The plants were uprooted and immediately transported on ice in a constant-temperature box to the laboratory. The rice plants were shaken to remove the loose soil around the roots, and the remaining soil was collected and thoroughly homogenized as rhizosphere soil samples (Dong et al., 2021). Rhizosphere soil samples collected in 2015 were divided into two parts: part one was air dried, passed through a 2 mm sieve and sent to the Qiyang Red Soil Experimental Station (26°45′N, 111°52′E, Hunan Province, China) for soil chemical analyzes (Sun et al., 2015; Wang W. et al., 2016); part two was stored at −80°C for soil DNA extraction. Soil pH was determined with a glass electrode at a soil/water ratio of 1:2.5. Organic matter (OM) was determined by the potassium dichromate volumetric method (Schollenberger, 1931). Total N (TN) was determined by the Kjeldahl digestion method (Bremner and Mulvaney, 1982). Total P (TP) and total K (TK) were determined by isolated by HF–HClO_4_ (Jacson, 1958), followed by molybdenum-blue colorimetry and flame photometry, respectively. Alkaline hydrolysis N (AN) was measured using the NaOH pervasion method (Bao, 2000). Available P (AP) was extracted with sodium bicarbonate and determined by the molybdenum blue method (Schollenberger, 1931). Available K (AK) was extracted with ammonium acetate and determined by flame photometry (Dahnke, 1988). Nitrate-nitrogen (NO_3_-N) and ammonium-nitrogen (NH_4_-N) were extracted with KCl and analyzed using a continuous flow analytical system (SA1000, Skalar, Netherlands; Wang J. et al., 2019). All chemical analyzes were performed in triplicate for each soil sample. Next, all rice roots of a specific sample were washed with distilled water, cut into 2 ~ 3 cm segments and thoroughly mixed. Subsequently, 50 mg of roots was placed in a sterile Petri dish, sterilized with 75% ethanol for 1 min and 1% NaClO for 4 min, frozen in liquid nitrogen and then stored at −80°C. The last washing solution was plated on a TSA solid medium to detect whether the roots were completely disinfected. The sterilized root samples collected in 2015 were used for DNA extraction.

In 2016, rhizosphere soil and rice root samples were collected on 27 July (tillering stage), 12 September (heading stage) and 25 October (mature stage; Supplementary Figure S1B). For each time point, 4 blocks (blocks I, II, III and IV) were set up for each fertilization regime, and 3 rice plants were randomly collected from the sampling region of each replicate plot and then mixed as a biological replicate. Thus, there were 4 biological replicates per fertilization regime. The preparation methods of rhizosphere soil and root samples in 2016 were in accordance with the methods in 2015. Rhizosphere soil samples collected in 2016 were divided into three parts: part one was used for soil chemical analyzes, and the other two parts were stored at −80°C separately for soil DNA and RNA extraction. Moreover, 50 mg and 100 mg of sterilized root samples were used for DNA and RNA extraction, respectively.

### DNA and RNA extraction and *nifH* Illumina sequencing

According to the manufacturer’s instructions, soil DNA and RNA were extracted using the FastDNA™ SPIN Kit for Soil (MP Biomedical, Irvine, CA, United States) and RNA PowerSoil^®^ Total RNA Isolation Kit (Mo Bio Laboratories, Inc., Carlsbad, CA, United States), respectively. Root DNA and RNA were extracted by the PowerPlant^®^ DNA Isolation kit (Mo Bio) and the RNApure Plant Kit (PD Biotech, Shanghai, China), respectively. The quality of extracted DNA and RNA was evaluated on a 1% agarose gel. Reverse transcription was performed using the PrimeScript™ RT reagent Kit with gDNA Eraser (Takara, Dalian, China).

DNA-derived and RNA-derived *nifH* gene Illumina sequencing was performed to elucidate the total and active diazotrophic community composition, respectively. The primers nifH1 (5′-barcode-TGYGAYCCNAARGCNGA-3′) and nifH2 (5’-ADNGCCATCATYTCNCC-3′) were used to amplify the *nifH* gene (Zehr and Mcreynolds, 1989). The barcodes were eight-base sequences unique to each sample. PCRs were performed in triplicate to minimize PCR bias and contained 4 μl of 5 × FastPfu Buffer, 2 μl of 2.5 mM dNTPs, 0.8 μl of each primer (5 μM), 0.4 μl of FastPfu Polymerase and 10 ng of template DNA or complementary DNA (Hong et al., 2015). Purified PCR products were pooled in equimolar amounts and paired-end sequenced (2 × 250 bp) on the Illumina MiSeq platform (Biozeron, Shanghai, China) according to standard protocols. Due to improper operation of root RNA extraction at the tillering stage, reverse transcription and *nifH* gene sequencing were only performed on the root RNA samples at the heading and mature stages. In total, 120 rhizosphere soil DNA samples, 48 rhizosphere soil RNA samples, 120 root DNA samples and 32 root RNA samples were collected for subsequent *nifH* gene sequencing. After removing one root DNA sample and one root RNA sample with low sequencing qualities, a total of 318 samples were obtained for further sequence processing.

### Sequence processing

Raw FASTQ files were quality filtered with the following criteria: (1) The bases of each read with quality scores <20 were discarded. Only the reads with perfectly matched barcodes, primers with <2 nucleotide mismatches and containing no ambiguous characters were retained. (2) Then, the barcodes and primers were deleted. (3) The remaining forward and reverse reads with at least a 10-bp overlap were combined into a single sequence using FLASH. Combined sequences of less than 250 bp were discarded. The quantified reads were translated into protein sequences, and potential frameshifts were corrected using the FrameBot program^1^ and the corresponding FunGene database^2^ (min length = 100 amino acids, hmm = 50%) as a reference (Fish et al., 2013). Reads with in-frame stop codon(s) were manually removed. Then, singletons were removed from the protein reads, and the remaining reads were dereplicated and clustered into operational taxonomic units (OTUs) using the UPARSE pipeline at 90% identity (Lema et al., 2012; Edgar, 2013). Chimeric sequences were then identified and removed using UPARSE in *de novo* mode. Finally, OTUs that contained one sequence were removed. To assign putative taxonomy, representative sequences were aligned to the closest-match sequences of the FunGene database by BLASTp (Zhang B. et al., 2015).

### Statistical analyzes

Richness (Chao1), evenness (Shannon), and phylogenetic diversity (PD) indices were calculated by Mothur software after sequences were normalized. The Venn diagram was analyzed using the online software, Venny.^3^ Diazotrophic *β*-diversity was assessed by computing the Bray–Curtis distance matrix and then ordinated using the nonmetric multidimensional scaling (NMDS) ordinations. Differences in the *β* diversity of diazotrophs were evaluated by analysis of similarity (ANOSIM) based on Bray–Curtis distances in R software (Version 4.0.2) using the vegan library (Clarke, 1993). Extended error bar plots were generated by the Statistical Analysis of Metagenomic Profiles (STAMP)^4^ to filter the root-enriched biomarkers (Parks et al., 2014). Welch’s two-sided *t*-test and the CI method (DP Welch’s inverted, Benjamini-Hochberg false discovery rate (FDR) correction, and *p*-value filter of 0.05) were applied to select genera differing significantly between the rhizosphere soil and root samples. The relative contributions of growth period and fertilization regime to diazotrophic community dissimilarity were tested with permutational multivariate analysis of variance (PERMANOVA) using the Adonis function from the R package “vegan.” PERMANOVA based on Bray–Curtis distances was performed at the OTU level (permutations = 999).

The parametric (Student’s *t*) test or nonparametric (Wilcoxon’s rank-sum) test was used to compare the Bray–Curtis distances between the diazotrophic communities of samples collected at the mature stage and samples collected at the other growth periods. Sankey diagrams were constructed based on D3.js (v.5.14.2, d3js.org; Ren et al., 2020) to illustrate the flows over time in diazotrophic communities and track the dynamics of individual OTUs among total rhizosphere and endophytic diazotrophic communities. The linear discriminant analysis (LDA) effect size algorithm (LEfSe) analysis was performed (Wilcoxon value of *p* < 0.05, logarithmic LDA score > 3) to identify biomarkers whose relative abundance significantly changed during rice growth (Segata et al., 2011). The differences in the relative abundances of these identified genera at different growth periods are displayed *via* heatmaps. Bar plots, box plots and bubble plots were drawn using the “ggplot2” package, and heatmaps was drawn using the “pheatmap” package in R v.4.0.2. The diazotrophic co-occurrence networks at each sampling point were constructed with the “psych” package in R v.4.0.2 based on the OTU tables at a 0.01% relative abundance threshold (Ye et al., 2020), and only robust (Spearman coefficient *r* > 0.65 or *r* < −0.65) and statistically significant (FDR corrected *p* < 0.01) correlations were kept. The networks were visualized in Gephi (Bastian et al., 2009). One-way analysis of variance (ANOVA) was conducted using SPSS 19.0 (SPSS Inc., United States). *Post hoc* comparisons were performed using Tukey’s HSD test (*p* < 0.05).

## Results

### Soil chemical properties

Soil pH, OM, TN, TP, TK, AN, AP, AK, NO_3_-N and NH_4_-N were all significantly affected by fertilization regimes (*p* < 0.05, Supplementary Table S1). The pH values of CK were highest (7.45 ± 0.22), followed by those of NPKM (7.08 ± 0.18), NPK (6.95 ± 0.31), and NPKS (6.74 ± 0.33). The pH values of NPKM were not significantly different from those of NPK and NPKS in 2015 (*p* > 0.05) but were significantly different from those in 2016 at the tillering and mature stages (*p* < 0.05). In 2015 and 2016, compared with CK, three fertilization treatments (NPK, NPKM and NPKS) all increased the contents of OM at three growth periods and the contents of TN, TP, AN and AP at the heading and mature stages, except that the content of AN of the NPK treatment was lower than that of CK at the heading stage in 2016. Among four fertilization regimes, the contents of OM, TN, TP, AN, AP and NO_3_-N of the NPKM treatment were highest at three growth periods, except that the contents of AN of the NPKM treatment were lower than those of the NPKS treatment at the heading stage in 2015 and 2016.

### The ***α***-diversity and community composition of total and active diazotrophs

**Figure 1:**
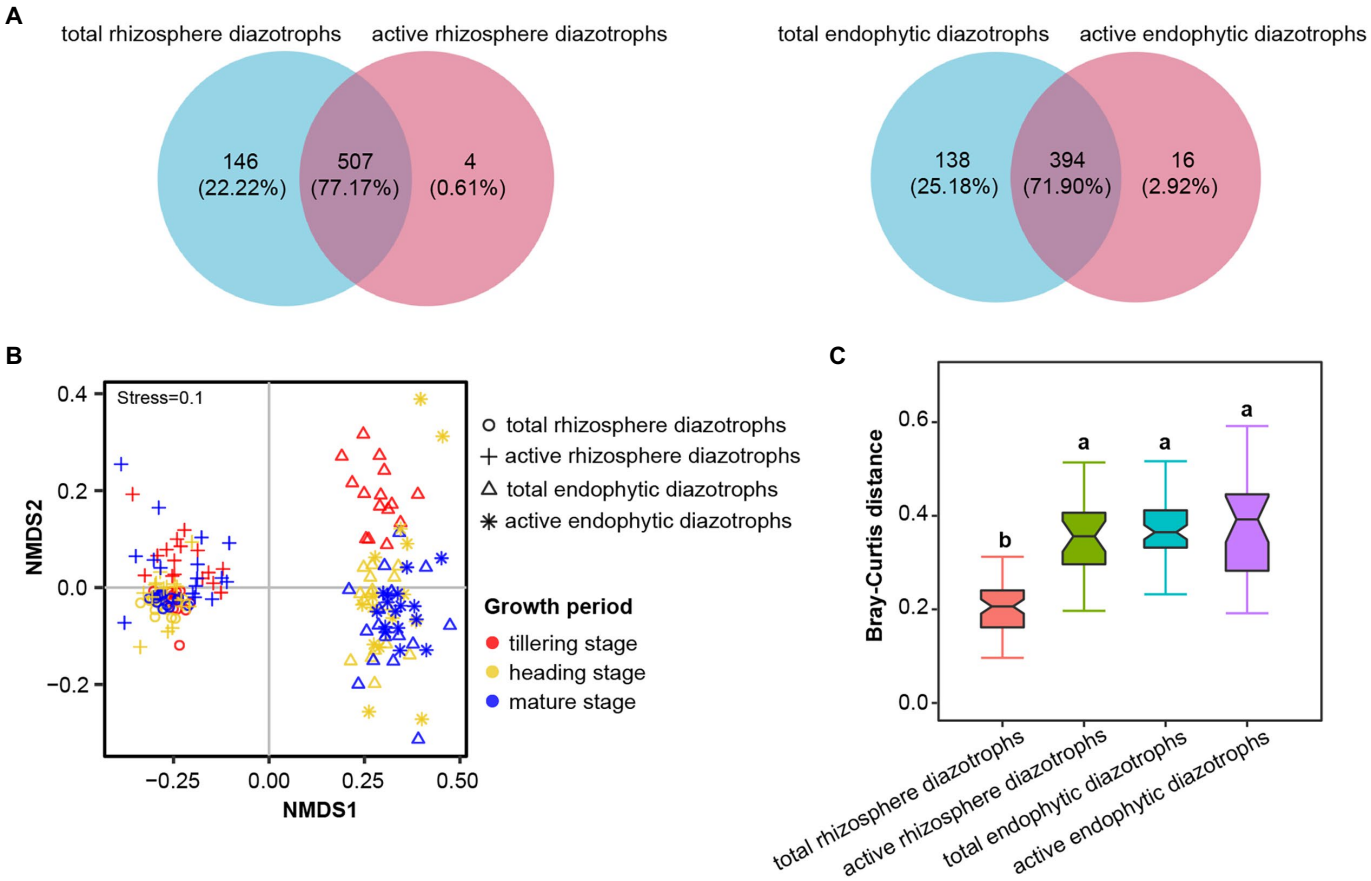
Effects of microhabitat on the community composition of total (DNA-derived) and active (RNA-derived) root-associated diazotrophs in 2016. **(A)** Venn diagrams showing the unique and shared OTUs between DNA and RNA samples in rhizosphere soil (at the tillering, heading and mature stages) and roots (at the heading and mature stages) in 2016. **(B)** Nonmetric multidimensional scaling (NMDS) ordinations based on the Bray–Curtis distance matrix of the total and active diazotrophic communities in rhizosphere soil and root samples in 2016. **(C)** Bray–Curtis distances of diazotrophic communities between soil DNA samples, soil RNA samples, root DNA samples or root RNA samples in 2016. Different lowercase letters indicate significant differences between these libraries (*p* < 0.05, Tukey’s HSD test).

After normalization, each DNA or RNA sample had 22,552 sequences. These sequences were clustered into 706 OTUs at 90% identity, with 643 bacterial OTUs (91.08%), 27 archaeal OTUs (3.82% of reads) and 36 unclassified OTUs (5.10% of reads). In Figure 1A, a total of 507 OTUs (77.17%, representing 99.86% of sequences) were shared between total (DNA-derived) and active (RNA-derived) rhizosphere diazotrophs, and 394 OTUs (71.90%, representing 99.88% of sequences) were shared between total and active endophytic diazotrophs in 2016, indicating that the total and active diazotrophic community composition presented considerable overlap. The Chao1 and PD indices of active diazotrophs (RNA samples) in rhizosphere soil and rice roots were significantly lower than those of the corresponding total diazotrophs (DNA samples) at the same growth period (*p* < 0.05, Supplementary Table S2). Taxonomic classification showed that fourteen phyla were identified by pooling the *nifH* DNA and RNA sequences, and the predominant phylum was Proteobacteria (Supplementary Figure S2). The proportions of Proteobacteria in the total and active rhizosphere diazotrophic communities (56.97 and 60.72%, respectively) were lower than those in the total and active endophytic diazotrophic communities (76.10 and 72.48%, respectively).

### Distinct communities of rhizosphere and endophytic diazotrophs

The *α*-diversity indices (Chao1, Shannon and PD indices) of total rhizosphere diazotrophs significantly higher than those of total endophytic diazotrophs in 2015 and 2016 (*p* < 0.05; Supplementary Table S2). The Chao1, Shannon and PD indices of active rhizosphere diazotrophs were significantly higher than those of active endophytic diazotrophs at the heading stage (*p* < 0.05), but the Chao1 and PD indices of active rhizosphere and endophytic diazotrophs were not significantly different at the mature stage in 2016 (*p* > 0.05). NMDS ordinations (Figure 1B; Supplementary Figure S3) and ANOSIM (*R* > 0.959, *p* < 0.001; Supplementary Table S3) showed strong significant differences in rhizosphere and endophytic diazotrophic communities at the DNA and RNA levels. The Bray–Curtis distances of the total rhizosphere diazotrophic communities were significantly less than those of the total endophytic, active rhizosphere and active endophytic diazotrophic communities in 2016 (*p* < 0.05, Figure 1C).

**Figure 2:**
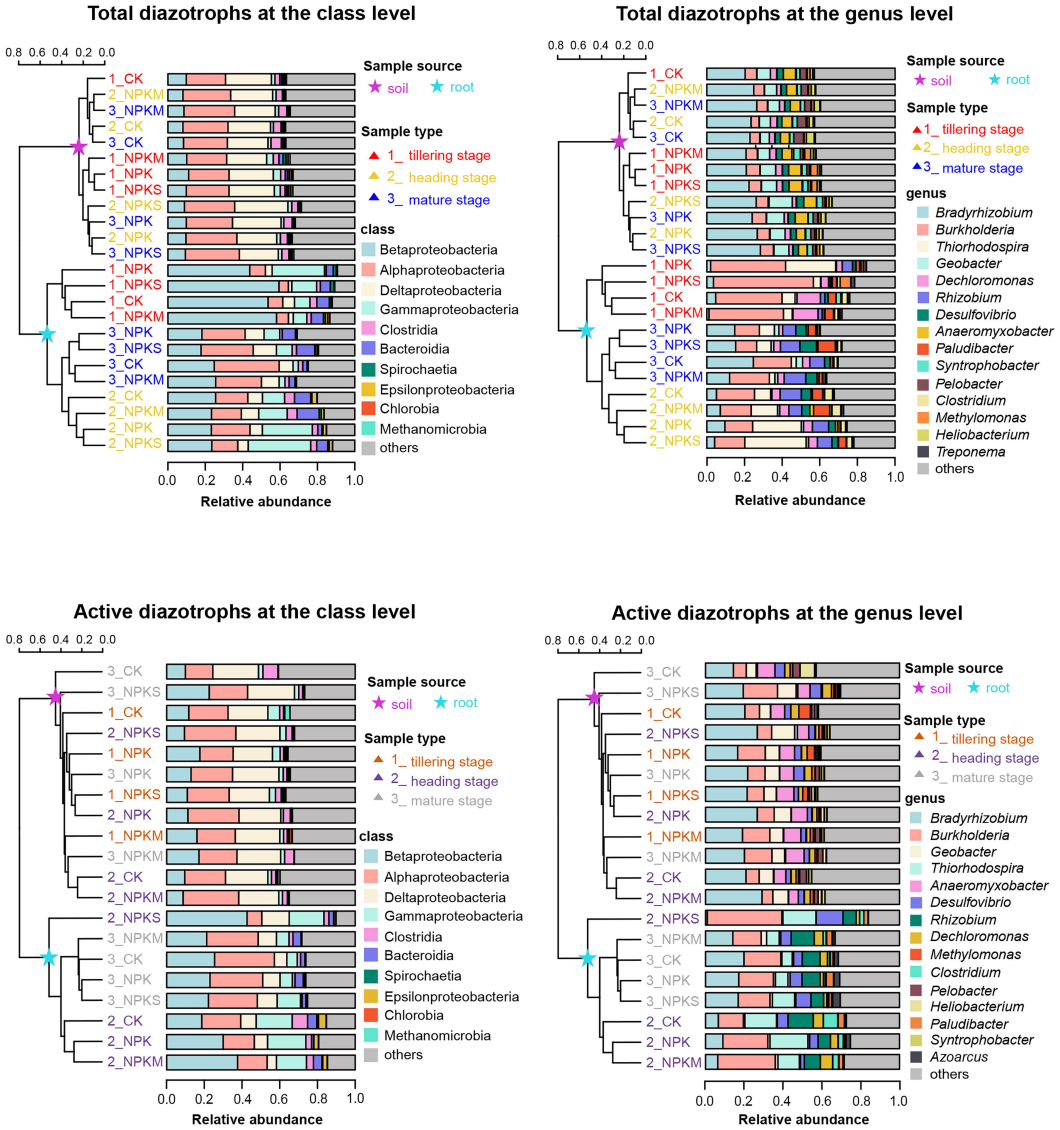
Community composition of total and active diazotrophs in rhizosphere soil and root samples at the class and genus levels in 2016. The unweighted pair group method with arithmetic mean (UPGMA) dendrogram was constructed based on Jaccard’s similarity coefficient calculated from the OTU table.

As shown in Figure 2; Supplementary Figure S4, the top three classes of total and active rhizosphere diazotrophic communities were Alphaproteobacteria (25.29 and 21.96%, respectively), Deltaproteobacteria (20.65 and 22.76%, respectively) and Betaproteobacteria (8.83 and 13.17%, respectively), and the three dominant classes of total and active endophytic diazotrophic communities were Betaproteobacteria (34.95 and 27.26%, respectively), Gammaproteobacteria (18.51 and 13.02%, respectively) and Alphaproteobacteria (15.37 and 21.91%, respectively). The proportions of “others” (mainly unclassified or uncultured categories) in soil DNA and RNA samples (35.61%) were higher than those in root DNA and RNA samples (18.01%). At the genus level, *Bradyrhizobium* (23.82%), *Geobacter* (7.67%), *Burkholderia* (6.95%), *Anaeromyxobacter* (4.78%), *Desulfovibrio* (2.93%) and *Dechloromonas* (2.84%) were the top six genera of total and active rhizosphere diazotrophic communities, but for total and active endophytic diazotrophic communities, the prevailing genera were *Burkholderia* (25.61%), *Thiorhodospira* (9.47%), *Bradyrhizobium* (8.92%), *Rhizobium* (6.22%) and *Tolumonas* (6.12%).

STAMP software was used to identify root-enriched diazotrophs (relative abundance >0.1%) whose relative abundances in rice root samples were significantly higher than those in rhizosphere soil samples (*p* < 0.05). In Supplementary Figure S5, the relative abundances of *Burkholderia*, *Thiorhodospira* and *Rhizobium* in root DNA and RNA samples (25.61, 9.47 and 6.22%, respectively) were significantly higher than those in soil DNA and RNA samples (6.95, 0.24 and 0.15%, respectively) at most sampling points (*p* < 0.05). However, *Bradyrhizobium*, *Geobacter* and *Anaeromyxobacter* had significantly lower relative abundances in root DNA and RNA samples (8.92, 1.57 and 0.58%, respectively) than in soil DNA and RNA samples (23.82, 7.67 and 4.78%, respectively; *p* < 0.05).

### Diazotrophic communities were shaped more strongly by the growth period than by the fertilization regime

As shown in Supplementary Table S4, the rice growth period had a greater influence on the *α*-diversity indices (Chao1, Shannon and phylogenetic diversity indices) of total endophytic diazotrophs (PERMANOVA, *R*^2^ ≥ 0.51, *p* < 0.001) than those of total rhizosphere diazotrophs (*R*^2^ ≤ 0.26, *p* < 0.001). The *α*-diversity indices of the total rhizosphere diazotrophic communities among the three growth stages were not significantly different in 2015 and 2016 (*p* > 0.05), while the indices of the total endophytic diazotrophic communities significantly increased from the tillering stage to the heading stage (*p* < 0.05; Supplementary Table S2). From the heading stage to the mature stage, the *α*-diversity indices of active rhizosphere diazotrophic communities significantly decreased (*p* < 0.05), while those of active endophytic diazotrophic communities showed no significant difference (*p* > 0.05).

**Figure 3:**
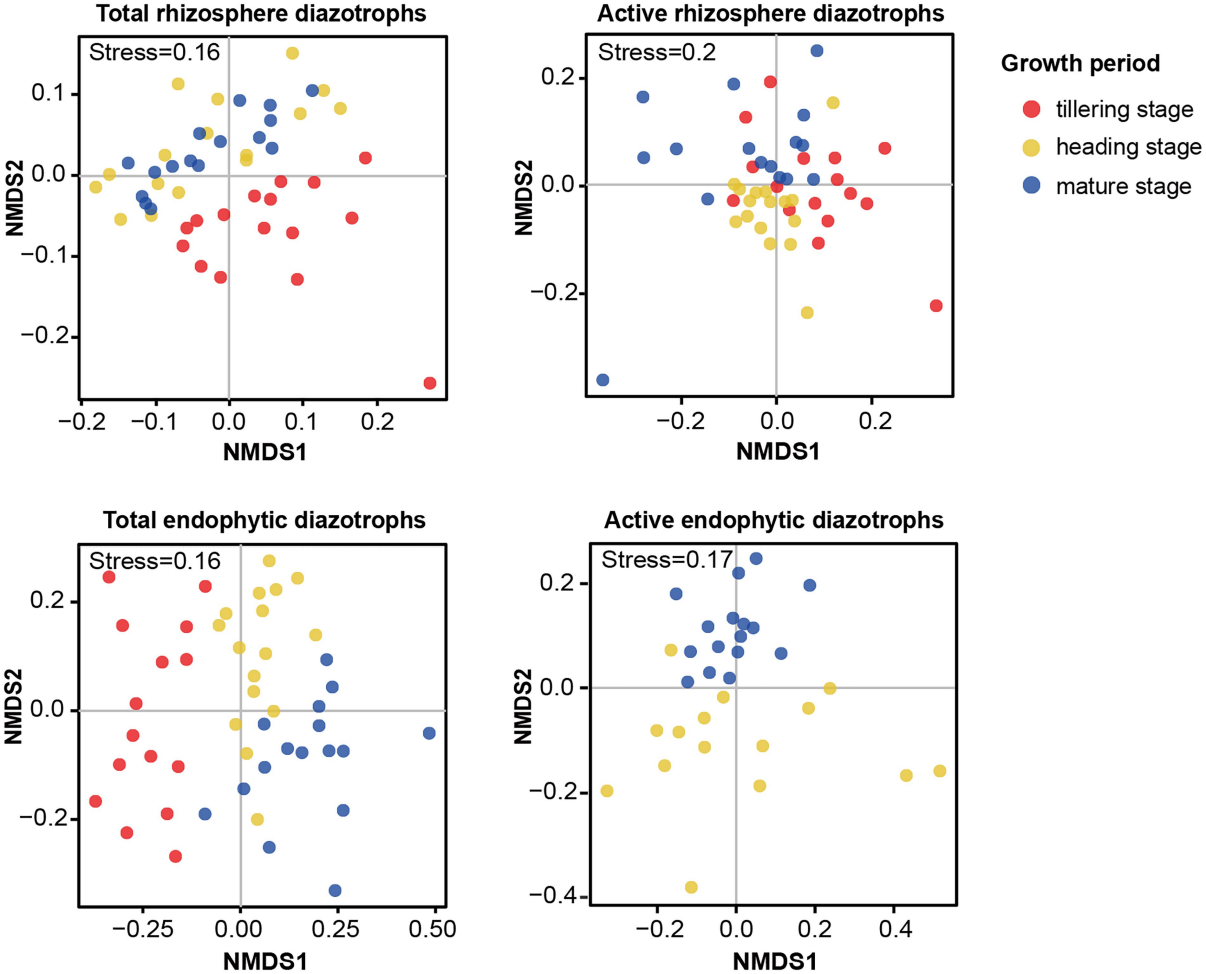
Effects of growth period on the community composition of total and active root-associated diazotrophs in 2016. NMDS ordinations based on the Bray–Curtis distance matrix of diazotrophic communities in each microhabitat at the DNA and RNA levels in 2016.

The NMDS plots (Supplementary Figure S6) and PERMANOVA analysis (Table 1) indicated that compared with the fertilization regime, the growth period was a primary driver of the total and active diazotrophic community composition in each microhabitat in 2015 and 2016. For instance, the variation in the total rhizosphere diazotrophic community composition was mainly explained by the plant growth period (*R*^2^ = 0.22, *p* < 0.001) then by the fertilization regime (*R*^2^ = 0.11, *p* < 0.001) (Table 1). The endophytic diazotrophic community was more distinctly separated according to growth period than the rhizosphere diazotrophic community, at the DNA and RNA levels (Figure 3; Supplementary Figure S7). Moreover, the growth period had a stronger effect on the community of total endophytic diazotrophs (*R*^2^ = 0.33, *p* < 0.001) than that of total rhizosphere diazotrophs (*R*^2^ = 0.22, *p* < 0.001), but fertilization practice had a weaker effect on the community of total endophytic diazotrophs (*R*^2^ = 0.05, *p* < 0.001) than that of total rhizosphere diazotrophs (*R*^2^ = 0.11, *p* < 0.001) (Table 1). Similarly, growth period had a stronger effect on the community of active endophytic diazotrophs (*R*^2^ = 0.17, *p* < 0.001) than that of active rhizosphere diazotrophs (*R*^2^ = 0.15, *p* < 0.001), but fertilization practice had a weaker effect on the community of active endophytic diazotrophs (*R*^2^ = 0.14, *p* < 0.001) than that of active rhizosphere diazotrophs (*R*^2^ = 0.1, *p* > 0.05).

**Table 1:** Effects of growth period, fertilization regime and year on the community composition of rhizosphere and endophytic diazotrophs.

Factor	Total rhizosphere diazotrophs		Total endophytic diazotrophs		Active rhizosphere diazotrophs		Active endophytic diazotrophs	
	*R* ^2^	Pr (>*F*)	*R* ^2^	Pr (>*F*)	*R* ^2^	Pr (>*F*)	*R* ^2^	Pr (>*F*)
Growth period (Gr)	0.22	<0.001	0.33	<0.001	0.15	<0.001	0.17	<0.001
Fertilization (Fe)	0.11	<0.001	0.05	<0.001	0.14	<0.001	0.1	0.203
Year (Ye)	0.19	<0.001	0.06	<0.001	-	-	-	-
Gr × Fe	0.04	0.004	0.09	<0.001	0.14	0.002	0.1	0.276
Gr × Ye	0.11	<0.001	0.08	<0.001	-	-	-	-
Fe × Ye	0.02	0.008	0.02	0.045	-	-	-	-
Gr × Fe × Ye	0.03	0.089	0.04	<0.001	-	-	-	-

Permutational multivariate analysis of variance (PERMANOVA) based on Bray–Curtis distances was performed at the OTU level (permutations = 999). The larger the value of *R*^2^ (the ratio of group variance to total variance), the more significant the differences were among the growth periods and fertilization regimes.

### Temporal dynamics of diazotrophic community composition

**Figure 4:**
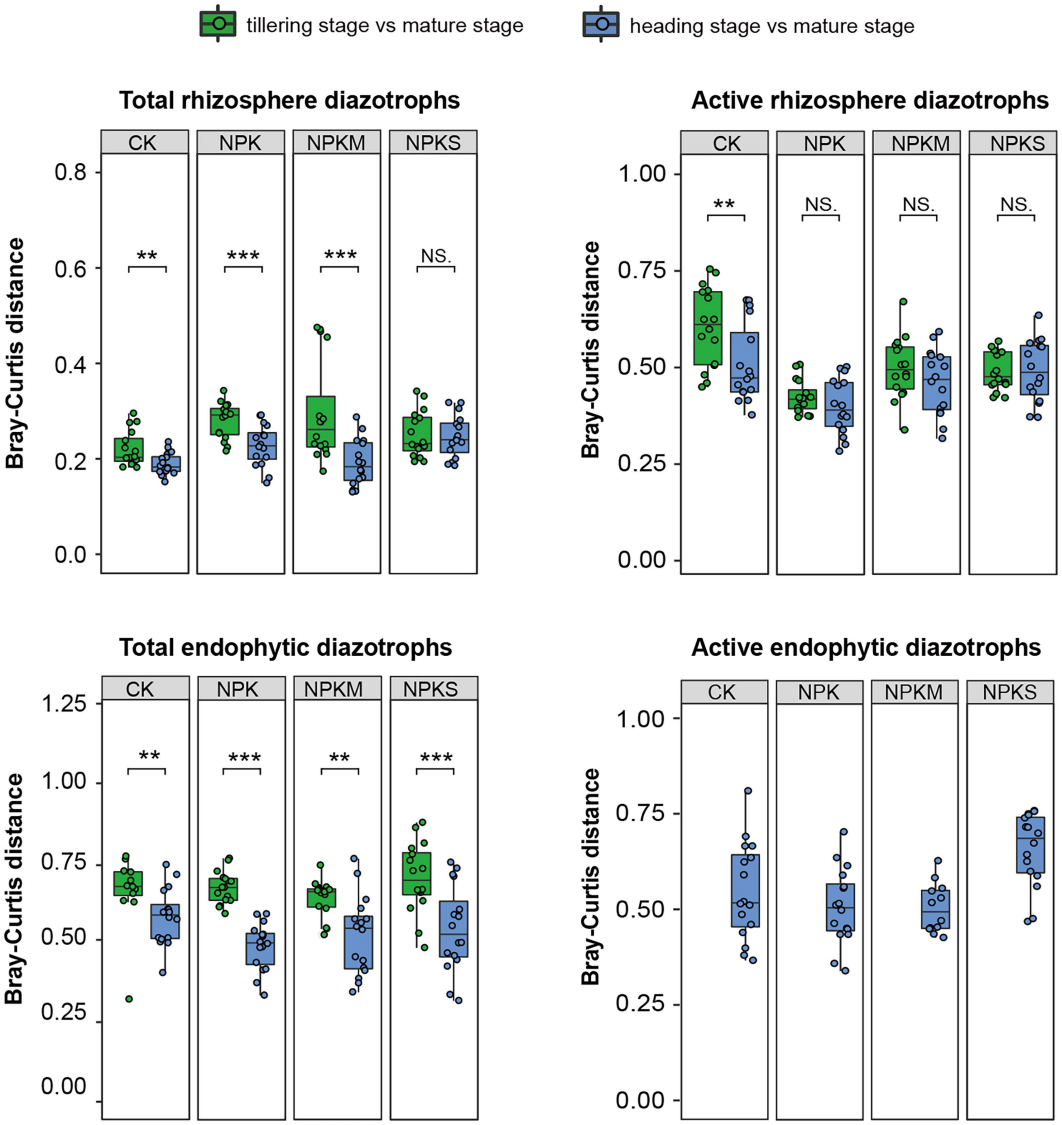
Bray–Curtis distances between the diazotrophic communities of samples collected at the mature stage and samples collected at the other growth periods in each microhabitat at the DNA and RNA levels in 2016. Significance level: *p* ≥ 0.05, ns; *p* < 0.05, ^*^p < 0.01, ^**^p < 0.001, ^***^(Student’s *t*-test or Wilcoxon’s rank-sum test).

In 2015 and 2016, the Bray–Curtis distances of the total endophytic diazotrophic communities between the heading stage and the mature stage under every fertilization regime (green boxes, 0.49 ± 0.09) were lower than those between the tillering stage and the mature stage (blue boxes, 0.70 ± 0.08; Figure 4; Supplementary Figure S8A). These results indicated that the Bray–Curtis distances of the total endophytic diazotrophic communities decreased, and the total endophytic diazotrophic communities gradually approached similarity during rice growth. However, this pattern was not obvious within the total and active rhizosphere diazotrophic communities. For example, the Bray–Curtis distances of the total rhizosphere diazotrophic communities between the heading and mature stages under every fertilization regime in 2015 and 2016 (0.30 ± 0.08) were lower than those between the tillering and mature stages (0.33 ± 0.07), but the Bray–Curtis distances of the total rhizosphere diazotrophic communities between the heading and mature stages (0.38 ± 0.02) were higher than those between the tillering and mature stages (0.32 ± 0.02) under the CK treatment (Figure 4; Supplementary Figure S8A). Furthermore, the Bray–Curtis distances of the active rhizosphere diazotrophic communities between the heading and mature stages (0.46 ± 0.09) were slightly lower than those between the tillering and mature stages (0.50 ± 0.10) (Figure 4).

**Figure 5:**
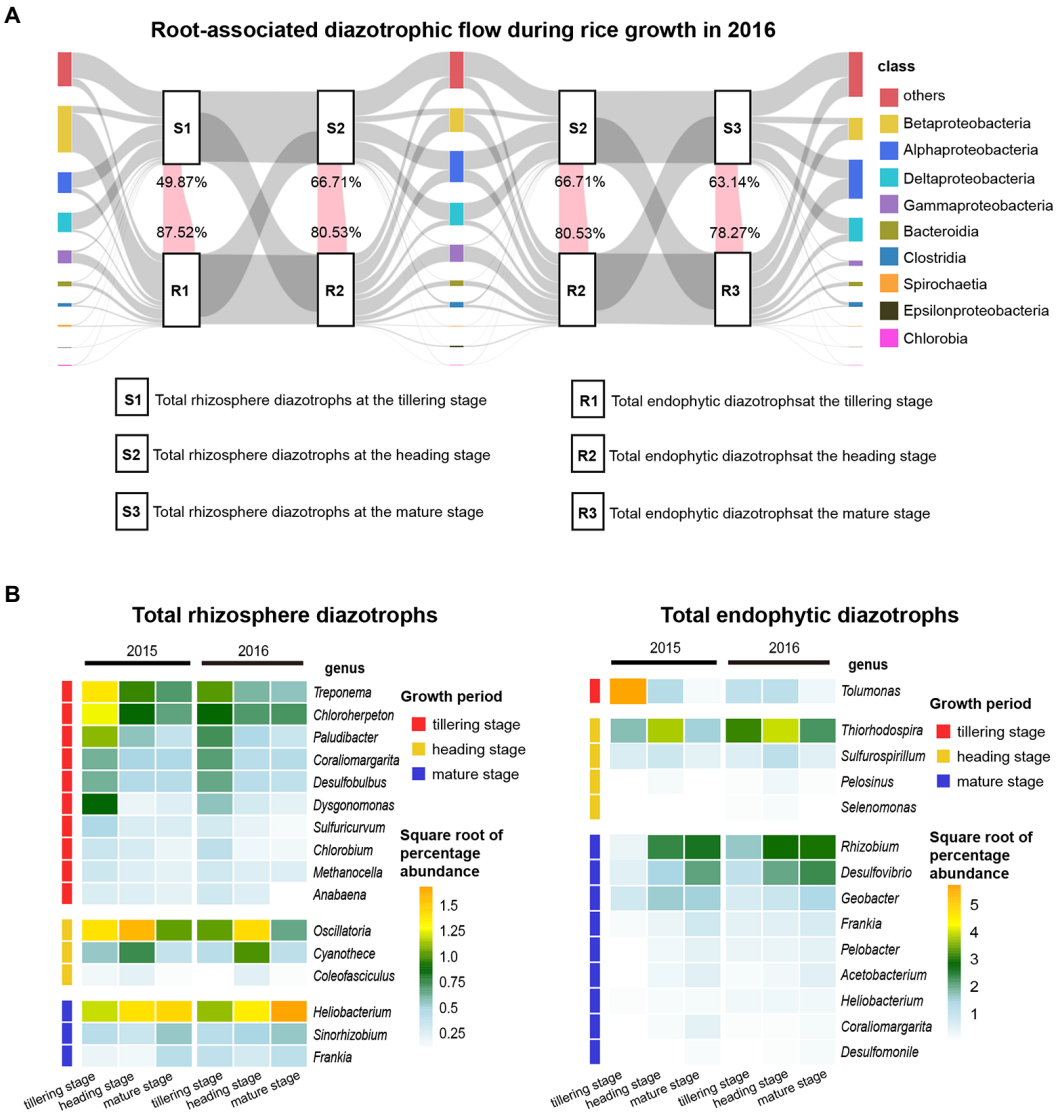
Temporal dynamics of total diazotrophic community composition in rhizosphere soil and root samples. **(A)** Root-associated diazotrophic flows during rice growth at the class level in 2016. The far left, middle and far right bars indicate the community composition of rhizosphere and endophytic diazotrophs at the tillering, heading and mature stages, respectively. Different color bars indicate different classes, and the heights of these bars indicate the relative abundances of the classes. Each class and each sample source (total rhizosphere diazotrophs or total endophytic diazotrophs) at the same sampling point were connected using gray bands as background. Pink flows represent variations in the relative abundance of OTUs shared by total rhizosphere and endophytic diazotrophs at each sampling point. The numbers at the top of the pink flows indicate the percentages of the sequence numbers of these shared OTUs in the total sequence numbers of rhizosphere diazotrophs. The numbers at the bottom of the pink flows indicate the percentages of the sequence numbers of these shared OTUs in the total sequence numbers of endophytic diazotrophs. **(B)** Heatmap displaying the changes in the relative abundances of discriminating genera throughout rice growth in soil and root DNA samples. The color of each large rectangle on the right indicates the square root of the percentage abundance of the genera. The small rectangles on the far left indicate the discriminating genera in soil and root DNA samples at different growth periods.

Tracking the changes in total diazotrophic communities at the class level in 2015 (Supplementary Figure S8B) and 2016 (Figure 5A) revealed that the total rhizosphere and endophytic diazotrophic communities showed distinct temporal dynamics at the class level. For instance, the relative abundances of “others” (mainly unclassified or uncultured taxa) in the total rhizosphere diazotrophic communities changed slightly during rice growth, but the relative abundances of “others” in the total endophytic diazotrophic communities markedly increased during rice growth and became gradually closer to those in the total rhizosphere diazotrophic communities. The relative abundances of Betaproteobacteria in the total rhizosphere diazotrophic communities first decreased and then increased over time, but those in the total endophytic diazotrophic communities continued to decrease dramatically (Figure 2; Supplemental Figure S4). The relative abundances of Alphaproteobacteria and Deltaproteobacteria in the total rhizosphere diazotrophic communities changed slightly, but those in the total endophytic diazotrophic communities were obviously enriched (Figure 2; Supplementary Figure S4). Moreover, at the three sampling points (tillering, heading and mature stages) in 2016, the relative abundances of OTUs shared by the total rhizosphere and endophytic diazotrophs (pink flows) accounted for 49.87, 66.71 and 63.14% of the total rhizosphere diazotrophs and 87.52, 80.53 and 78.27% of the total endophytic diazotrophs, respectively. In 2015, the relative abundances of OTUs shared by the total rhizosphere and endophytic diazotrophs accounted for 30.53%, 58.16 and 73.26% of the total rhizosphere diazotrophs and 90.09, 86.09 and 79.20% of the total endophytic diazotrophs, respectively. These results revealed that the proportions of OTUs shared by the total rhizosphere and endophytic diazotrophs in rhizosphere diazotrophs increased gradually, while the proportions in endophytic diazotrophs decreased gradually.

Moreover, the distinguishing genera that significantly proliferated or were inhibited during the cultivation cycle were filtered using the LEfSe analysis and then displayed *via* heatmaps (Figure 5B). For total rhizosphere diazotrophs, the relative abundances of *Treponema*, *Chloroherpeton* and *Paludibacter* decreased gradually from the tillering stage to the mature stage, the relative abundances of *Oscillatoria* and *Cyanothece* first increased and then decreased significantly (*p* < 0.05), and the relative abundance of *Heliobacterium* increased gradually. For total endophytic diazotrophs, the relative abundance of *Tolumonas* peaked at the tillering stage, the relative abundance of *Thiorhodospira* and *Sulfurospirillum* first increased and then decreased significantly (*p* < 0.05), and the relative abundances of *Rhizobium*, *Desulfovibrio* and *Geobacter* increased gradually.

### Temporal dynamics of diazotrophic co-occurrence networks

**Figure 6:**
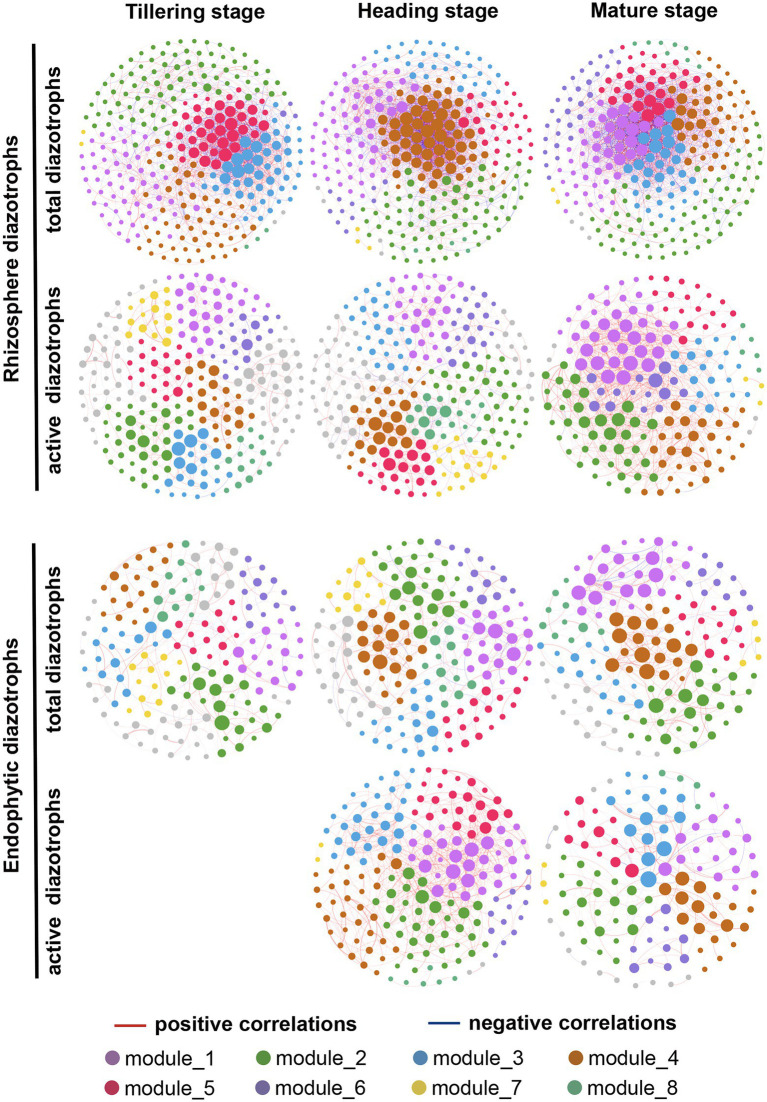
Co-occurrence networks of total and active root-associated diazotrophic communities at three growth periods in 2016. Different nodes represent different OTUs. Edges represent Spearman’s correlation relationships. Red solid lines show strong (Spearman’s correlation coefficient *r* > 0.65) and significant (*p* < 0.01) positive correlations between the nodes. Blue solid lines show strong (Spearman’s correlation coefficient *r* < −0.65) and significant (*p* < 0.01) negative correlations between the nodes. The size of each point represents the node’s weighted degree. Nodes in the eight largest modules are marked with different colors, while other nodes are marked with gray.

The network structures of total and active diazotrophs in rhizosphere soil and rice roots changed obviously over time (Figure 6; Supplementary Figure S9). As shown in Table 2, the edge numbers, average clustering coefficient (avgCC) and average degree (avgK) values of the total rhizosphere, active rhizosphere, total endophytic and active endophytic diazotrophic co-occurrence networks all increased from the tillering stage to the heading stage in 2015 and 2016. In the total rhizosphere and endophytic diazotrophic networks, the edge numbers and avgK values exhibited remarkable decreases from the heading stage to the mature stage in 2015 but showed slight differences in 2016. For instance, from the heading stage to the mature stage, the edge numbers and avgK values in the total rhizosphere diazotrophic networks decreased by 40.46 and 45.96%, respectively, in 2015, but these indices increased by 1.32 and 7.01%, respectively, in 2016. From the heading stage to the mature stage in 2016, the edge numbers and avgK values increased by 59.88 and 126.39%, respectively, in the active rhizosphere diazotrophic networks and decreased 75.64 and 65.35%, respectively, in the active endophytic diazotrophic networks.

**Table 2:** Topological properties of the co-occurrence networks of total and active diazotrophs in the rhizosphere soil and rice roots during three growth periods.

Network metrics	Soil									Root							
	DNA samples in 2015			DNA samples in 2016			RNA samples in 2016			DNA samples in 2015			DNA samples in 2016			RNA samples in 2016	
	T	H	M	T	H	M	T	H	M	T	H	M	T	H	M	H	M
Number of nodes	209	175	193	299	282	267	251	248	175	61	81	81	167	168	148	176	124
Number of edges	610	954	568	1797	2,352	2,383	430	648	1,036	65	105	76	260	361	344	780	190
Average path length (APL)	3.96	3.38	4.04	3.51	3.35	3.33	5.56	4.59	3.14	5.00	5.49	4.37	6.17	4.62	4.14	3.50	5.03
Graph density	0.03	0.06	0.03	0.04	0.06	0.07	0.01	0.02	0.07	0.04	0.03	0.02	0.02	0.03	0.03	0.05	0.03
Network diameter	12	13	10	10	9	11	15	13	9	13	13	12	18	11	9	10	14
Average clustering coefficient (avgCC)	0.35	0.49	0.43	0.35	0.41	0.39	0.27	0.29	0.44	0.29	0.32	0.24	0.25	0.31	0.34	0.39	0.25
Average degree (avgK)	5.84	10.90	5.89	12.02	16.68	17.85	3.43	5.23	11.84	2.13	2.59	1.88	3.11	4.30	4.65	8.86	3.07
Modularity (M)	0.53	0.28	0.51	0.37	0.30	0.27	0.73	0.61	0.41	0.79	0.70	0.78	0.74	0.66	0.58	0.48	0.69
Number of positive edges	374	533	346	1,059	1,279	1,250	379	518	1,019	61	95	58	215	275	271	776	147
Percent of positive correlations	0.61	0.56	0.61	0.59	0.54	0.52	0.88	0.80	0.98	0.94	0.90	0.76	0.83	0.76	0.79	0.99	0.77

The sampling time points represent the rice tillering stage (T), heading stage (H) and mature stage (M).

According to the co-occurrence networks of total diazotrophs in 2015 and 2016, the node numbers, edge numbers and avgK values decreased from rhizosphere soil to rice roots at the same sampling point (Table 2), which was consistent with the *α*-diversity results (Supplementary Table S2). The ratios of positive correlations in the total rhizosphere diazotrophic networks (0.57 ± 0.04) were much lower than those in the total endophytic diazotrophic networks (0.83 ± 0.08). The edge number and avgK value of active diazotrophic communities increased from rhizosphere soil to roots at the heading stage but decreased at the mature stage. In addition, the active rhizosphere and endophytic diazotrophic networks had high ratios of positive correlations (≥0.77).

## Discussion

### Does the DNA-derived diazotrophic community represent the RNA-derived diazotrophic community?

DNA- and RNA-derived sequencing approaches can provide a broad overview of the present and metabolically active microflora in different environments, such as soil (Tang et al., 2017), marine (Cardoso et al., 2017) and biotic (Welsh et al., 2010) environments. Our study revealed that the OTUs shared by total and active rhizosphere diazotrophs accounted for 77.17% of the total OTUs in rhizosphere soil samples, and the OTUs shared by total and active endophytic diazotrophs accounted for 71.90% of the total OTUs in rice root samples (Figure 1A), indicating that the rhizosphere soil and rice roots are “hotspots” for diazotrophic activities. This is reasonable as the rhizosphere is known to be a “hotspot” of microbial ecology and the root–plant–soil interactions (Zhang R. et al., 2017).

In this study, significantly lower *α*-diversity indices were observed for the active diazotrophs than for the total diazotrophs in rhizosphere soil and rice roots. The differentiation in *α*-diversity at the DNA and RNA levels was also observed for the plankton-associated diazotrophs (Yang et al., 2019) and rice rhizosphere bacteria (Li et al., 2019). Moreover, compared with total rhizosphere diazotrophs, the community composition of active rhizosphere diazotrophs was more volatile. Previous study on plankton-associated diazotrophs showed that between-community variation in the active diazotrophic communities among different sites was significantly higher than that in the total diazotrophic communities (*p* < 0.001; Yang et al., 2019). Furthermore, our results showed the differences in the community composition and the topological properties of co-occurrence networks (e.g., edge numbers, avgCC and avgK) of total and active diazotrophs. Taken together, these evidences suggest that there is a discrepancy in the present and actually active diazotrophic communities between the rhizosphere soil and rice roots. Thus, we suggested that DNA-derived and RNA-derived analyzes of diazotrophic communities could be combined in future experiments on the diazotrophic community composition and functional properties.

### Community succession of rhizosphere and endophytic diazotrophs

Revealing the formation and succession processes of plant-associated diazotrophs is essential to advance fundamental understandings of the coevolution of plants and diazotrophs and sustainable applications of diazotrophs in agriculture in the future. In this study, compared with the fertilization regime, the growth period was the dominant factor affecting the *α*-diversity and community composition of total and active root-associated diazotrophs. Further, at both the DNA and RNA levels, the growth period had a greater effect on the endophytic diazotrophic communities than on the rhizosphere diazotrophic communities. Consistently, previous studies on sorghum rhizosphere soil and wheat bulk soil showed that *nifH* gene abundances and diazotrophic communities were more sensitive to temporal variation (seasonal variation) than to fertilization regime (Hai et al., 2009; Wang J. et al., 2016).

Temporal dynamics of microbial abundance, composition and function has been reported (Silva et al., 2013; Wang W. et al., 2019), but little is known about the succession process of rice endophytic diazotrophic communities. Our study was the first to analyze the succession patterns of rhizosphere and root endophytic diazotrophs using high-throughput sequencing in field-grown rice. Our results demonstrated that there was no significant difference in the *α*-diversity indices of total rhizosphere diazotrophs among three growth periods, while the *α*-diversity indices of total endophytic diazotrophs significantly increased from the tillering stage to the heading stage (*p* < 0.05). Moreover, the edge numbers and avgK of co-occurrence networks of total rhizosphere, total endophytic, active rhizosphere and active endophytic diazotrophs all increased from the tillering stage to the heading stage. Chaparro et al. (2014) found that the community compositions of rhizosphere bacteria at the bolting and flowering stages had no significant difference, but they were distinct from those at the seedling stage. In our study, the Bray–Curtis distances of the total rhizosphere diazotrophic communities were significantly less than those of the total endophytic diazotrophic communities, but the total rhizosphere and endophytic diazotrophic communities separately tended to be similar during rice growth. These results suggest that the rice rhizosphere and endophytic diazotrophs had different patterns of community succession, whereas eventually, their community compositions tended to be similar.

According to the two-stage enrichment model (Bulgarelli et al., 2013), we hypothesized that abundant diazotrophs recruited from paddy soil might colonize and reproduce in rice roots at the vegetative stage. We found supports for this hypothesis: the *α*-diversity indices (Chao1, Shannon and PD indices) of total endophytic diazotrophs significantly increased from the tillering stage to the heading stage, indicating that more taxa were detected at the heading stage as compared to the tillering stage. The proportions of OTUs shared by soil and root DNA samples in soil DNA samples clearly increased over time, and the proportions of the unclassified or uncultured diazotrophs in root DNA samples markedly increased and became closer to those in rhizosphere soil (Figure 5A; Supplementary Figure S8B). From these results, we speculated that after being transplanted into the field, rice plants continuously recruited new endophytic diazotrophs (including some unclassified or uncultured diazotrophs) from rhizosphere soil, resulting in increasing amounts of the shared OTUs in total rhizosphere diazotrophs. However, the relative abundances of the shared OTUs in total endophytic diazotrophs gradually decreased. We speculated that despite rhizosphere diazotrophs continuously colonizing in the roots, the growth of root-specific endophytic diazotrophs dominated in roots. Considering that root exudation increased with rice growth until panicle initiation and decreased from the flowering period to the mature stage (Aulakh et al., 2001), the community succession of rice endophytic diazotrophs is probably driven by root exudation. However, further studies are needed to investigate the regulatory mechanism of root exudates on endophytic diazotrophic communities.

### Host selection effect reduced diazotrophic diversity and network complexity

Microhabitats influenced the *α*-diversity, *β*-diversity and network structures of the microbiome in various plants (Roesch et al., 2008; Fan et al., 2018; Yao et al., 2019; Zhang et al., 2020). We found that rhizosphere diazotrophs were more diverse than endophytic diazotrophs. This result is consistent with that seen in maize, where the diazotrophs in rhizosphere soil had greater diversity than those in the roots and stems (Roesch et al., 2008). Our results demonstrated that Alphaproteobacteria and Betaproteobacteria were the dominant classes of the rhizosphere and endophytic diazotrophs, respectively. Previous studies showed that members of the Alphaproteobacteria and Betaproteobacteria were dominant in rice rhizosphere soil and roots (Wu et al., 2009; Shu et al., 2012; Ferrando and Scavino, 2015). Moreover, we selected some root-enriched diazotrophs, which accounted for 46.29% ± 14.93% of the total endophytic diazotrophs and 47.77% ± 13.01% of the active endophytic diazotrophs. Among them, *Rhizobium* and *Burkholderia* are common plant endophytic diazotrophs and have been used as plant growth-promoting bacteria (PGPB) and biocontrol bacteria (Gutiérrez-Zamora and Martínez-Romero, 2001; Weber et al., 2007; Govindarajan et al., 2008). It is widely thought that endophytic diazotrophs are capable of fixing N more efficiently than rhizosphere or rhizoplane diazotrophs (Prakamhang et al., 2009). Therefore, we speculate that these root-enriched diazotrophs play important roles in rice growth and development throughout the life cycle of rice.

In recent years, molecular ecological network analysis (MENA) has been widely used to characterize potential interactions such as mutualism, synergy, competition and predation among different species/populations within complex ecological environments (Deng et al., 2012; Morriën et al., 2017). Our study showed that at the DNA and RNA levels, the node numbers, edge numbers and avgK values of rhizosphere diazotrophic co-occurrence networks were greater than those of endophytic diazotrophic co-occurrence networks at the same sampling point (Table 2), indicating that rhizosphere diazotrophs formed larger and more complex networks than endophytic diazotrophs. This result further supported the idea that the host selection effect reduced bacterial network complexity (Xiong et al., 2020). The ratio of positive correlations in total rhizosphere diazotrophic networks was lower than that in total endophytic diazotrophs, suggesting that diazotrophs in rhizosphere soil had more competitive relationships than those in roots. In general, compared with the total endophytic diazotrophs, the total rhizosphere diazotrophs had higher *α*-diversity, more stable community composition, more complex network structure and more competitive relationships. This could be partly explained by the intensive selection pressures from the host and the specificity of the root microenvironment (Zheng and Gong, 2019; Xiong et al., 2020).

## Conclusion

In this study, DNA- and RNA-derived *nifH* sequencing approaches provided a broad overview of the spatial (microhabitat) and temporal (rice growth period) distribution of present and metabolically active root-associated diazotrophs. Our results demonstrate that there is a discrepancy between the *α*-diversity, *β*-diversity and co-occurrence networks of the total and active root-associated diazotrophic communities. From rhizosphere soil to rice roots, the *α*-diversity and network complexity of total diazotrophic communities decreased, but the ratio of positive correlations in total diazotrophic networks increased. The community composition of total and active root-associated diazotrophs is shaped predominantly by the growth period rather than by the fertilization regime. Moreover, the community composition of the total rhizosphere, active rhizosphere and total endophytic diazotrophs approached similarity separately over time. From the tillering stage to the heading stage, the *α*-diversity indices and network complexity of the total endophytic diazotrophic communities increased. Overall, our study provides novel insights into the temporal dynamics of root-associated diazotrophic diversity, co-occurrence networks and inters taxa interactions in field-grown rice.

## Data availability statement

The datasets presented in this study are publicly available at https://www.ncbi.nlm.nih.gov/bioproject, accession number PRJNA666552.

## Author contributions

ZC, HC, WR, and XL conceived the study. XL, WW, YC, and ZL collected samples. XL, XY, and WW performed the DNA and RNA extractions. XL and YW performed data analyzes. XL wrote the manuscript. ZC, XY, and YH revised the manuscript. All authors contributed to the article and approved the submitted version.

## Funding

This work was supported by the Natural Science Foundation of China (32170123), the Natural Science Foundation of China (32000101), and the Major State Basic Research Development Program of China (973 program, no. 2015CB150502).

## Conflict of interest

The authors declare that the research was conducted in the absence of any commercial or financial relationships that could be construed as a potential conflict of interest.

## Publisher’s note

All claims expressed in this article are solely those of the authors and do not necessarily represent those of their affiliated organizations, or those of the publisher, the editors and the reviewers. Any product that may be evaluated in this article, or claim that may be made by its manufacturer, is not guaranteed or endorsed by the publisher.

## Footnotes

^1^ http://fungene.cme.msu.edu/FunGenePipeline/

^2^ http://fungene.cme.msu.edu/

^3^ https://bioinfogp.cnb.csic.es/tools/venny

^4^ https://beikolab.cs.dal.ca/software/STAMP
