# Supplementary material for: Temporal dynamics of total and active root-associated diazotrophic communities in field-grown rice
Source: Front Microbiol. 2022 Oct 13;13:1016547. doi: 10.3389/fmicb.2022.1016547 (PMC9606772; doi:10.3389/fmicb.2022.1016547)
Supplement: Supplementary file 1 [file Data_Sheet_1.PDF]

Supplementary Material

Title: Temporal dynamics of total and active root-associated diazotrophic communities in field-grown rice

Running title: Rice root-associated diazotrophic communities

A. Supporting Tables

Table S1 Two years of soil chemical properties among the four fertilization regimes

Table S2 The α-diversity indices of rhizosphere and endophytic diazotrophic communities during three growth periods in Jintan

Table S3 ANOSIM tests for the differences in the community composition between rhizosphere and endophytic diazotrophs among three growth periods

Table S4 Effects of growth period, fertilization regime and year on the α-diversity indices of rhizosphere and endophytic diazotrophs

B. Supporting Figures

Figure S1 Field experimental design. **(A)** Field layout of the 68 plots (8×5 m^2 each) in a random block design of seventeen fertilization regimes with four replicates. The four fertilization regimes chosen in this study were as follows: no fertilization (CK), 100% chemical fertilizers (NPK), 50% chemical fertilizers plus 6000 kg/ha pig manure (NPKM) and 100% chemical fertilizers plus 8000 kg/ha crop straw (NPKS). **(B)** Sampling strategy. Rhizosphere soil samples and rice root samples were collected at the tillering, heading and mature stages of rice. In 2015, rhizosphere soil and root samples were collected for DNA extraction. In 2016, the rhizosphere soil and root samples were collected for DNA and RNA extraction, except the root samples collected at the tillering stage, which were only used for DNA extraction. In total, 120 rhizosphere soil DNA samples, 48 rhizosphere soil RNA samples, 120 root DNA samples and 32 root RNA samples were collected for subsequent *nifH* gene sequencing.

Figure S2 Bar plot showing the mean and standard deviation of the relative abundances of diazotrophic taxa at the phylum level across different sample types in 2015 and 2016. Euryarchaeota marked in red is one of the four phyla of the domain Archaea, and the other phyla belong to the domain bacteria.

Figure S3 Effects of microhabitat and growth period on the community composition of total rhizosphere and endophytic diazotrophs in 2015. NMDS ordinations based on Bray–Curtis distance matrix of the total diazotrophic communities in rhizosphere soil and root samples.

Figure S4 Community composition of total diazotrophs in rhizosphere soil and root samples at the class and genus levels in 2015. The UPGMA dendrogram was constructed based on Jaccard's similarity coefficient calculated from the OTU table.

Figure S5 A bubble plot showing changes in the relative abundances of the dominant genera (relative abundance of diazotrophs > 0.1%) in the rhizosphere soil and root samples. The size of each bubble represents the square root transformed relative abundance percentage of each genus in the root samples, and the color represents the change in the relative abundance of each genus in the root and rhizosphere soil samples. Abundance difference = square root of the relative abundance percentage of a genus in the root sample - square root of the relative abundance percentage of the genus in the corresponding rhizosphere soil sample. Red bubbles indicate genera whose relative abundances are significantly higher in roots than in rhizosphere soil ($P < 0.05$, abundance change > 0), and blue bubbles indicate genera whose relative abundances are significantly lower in roots than in rhizosphere soil ($P < 0.05$, abundance change < 0). Cluster analysis of the genera was performed based on Euclidean dissimilarities using the complete linkage method.

Figure S6 Effects of microhabitat and fertilization regime on the community composition of total and active root-associated diazotrophs in 2015 (A) and 2016 (B). NMDS ordinations based on Bray–Curtis distance matrix of diazotrophic communities among different fertilization regimes.

Figure S7 Effects of growth period on the community composition of total rhizosphere and endophytic diazotrophs in 2015. NMDS ordinations based on Bray–Curtis distance matrix of total diazotrophic communities in rhizosphere soil and root samples.

Figure S8 Temporal dynamics of total diazotrophic community composition in rhizosphere soil and root samples in 2015. **(A)** Bray–Curtis distances between the diazotrophic communities of soil DNA samples collected at the mature stage and samples collected at the other growth periods in 2015; the same analyses were performed in corresponding root DNA sample in 2015. Significance level: $P \geq 0.05$, ns; $P < 0.05$, *; $P < 0.01$, **; $P < 0.001$, *** (Student's *t*-test or Wilcoxon's rank-sum test). **(B)** Root-associated diazotrophic flows during rice growth at the class level in 2015. The far left, middle and far right bars indicate the community composition of rhizosphere and endophytic diazotrophs at the tillering, heading and mature stages, respectively. Different color bars indicate different classes, and the heights of these bars indicate the relative abundances of the classes. Each class and each sample source (total rhizosphere diazotrophs or total endophytic diazotrophs) at the same sampling point were connected using gray bands as background. Pink flows represent variations in the relative abundance of OTUs shared by soil and root DNA samples at each sampling point. The numbers at the top of the pink flows indicate the percentages of the sequence numbers of these shared OTUs in the total sequence numbers of rhizosphere diazotrophs. The numbers at the bottom of the pink flows indicate the percentages of the sequence numbers of these shared OTUs in the total sequence numbers of endophytic diazotrophs.

Figure S9 Co-occurrence networks of total rhizosphere and endophytic diazotrophic communities at three growth periods in 2015. Different nodes represent different OTUs. Edges represent Spearman's correlation relationships. Red solid lines show strong (Spearman's correlation coefficient $r > 0.65$) and significant ($P < 0.01$) positive correlations between the nodes. Blue solid lines show strong (Spearman's correlation coefficient $r < -0.65$) and significant ($P < 0.01$) negative correlations between the nodes. The size of each point represents the node's weighted degree. Nodes in the eight largest modules are marked with different colors, while other nodes are marked with gray.

Table S1 Two years of soil chemical properties among the four fertilization regimes

Year	Growth period	Fertilization regime	pH	OM (g/kg)	TN (g/kg)	TP (g/kg)	TK (g/kg)	AN (mg/kg)	AP (mg/kg)	AK (mg/kg)	NH_4-N (mg/kg)	NO_3-N (mg/kg)
2015	Tillering stage	CK	7.54±0.34 a	26.81±1.51 b	1.75±0.15 b	0.83±0.1 a	12.96±0.23 a	248.88±12.39 a	15.5±0.59 c	104.17±17.39 b	-	-
		NPK	7.23±0.14 b	27.19±2.95 b	1.72±0.05 b	0.80±0.18 a	12.08±0.64 ab	237.26±22.39 a	12.84±2.29 c	107.17±1.72 b	-	-
		NPKM	7.16±0.08 b	32.64±0.64 a	2.05±0.24 a	0.97±0.03 a	11.3±0.19 bc	258.04±28 a	33.19±4.08 a	141.83±16.04 a	-	-
		NPKS	7.05±0.04 b	30.35±1.77 a	1.88±0.07 ab	0.81±0.12 a	10.98±1 c	226.84±14.94 a	20.21±2.56 b	142±17.88 a	-	-
	Heading stage	CK	7.27±0.07 a	28.79±1.97 c	1.72±0.01 c	0.58±0.02 c	10.42±0.34 a	178.63±14.2 b	8.63±1.19 d	98.17±12.62 a	-	-
		NPK	7.03±0.14 b	30.78±1.59 bc	1.85±0.07 b	0.68±0.04 b	9.9±0.29 a	196.49±14.98 b	14.75±2.45 c	94.67±17.39 a	-	-
		NPKM	6.93±0.06 b	35.88±1.73 a	2.13±0.04 a	0.93±0.05 a	9.67±0.32 a	225.2±12.25 a	30.08±2.79 a	99.17±7.71 a	-	-
		NPKS	6.52±0.12 c	33.25±2.86 ab	2.07±0.09 a	0.72±0.05 b	10.53±1.08 a	237.3±21.52 a	19.69±3.65 b	106.33±15.25 a	-	-
	Mature stage	CK	7.51±0.16 a	27.21±1.41 b	1.66±0.06 c	0.52±0.03 c	9.51±0.51b	186.19±8.11 b	5.81±1.07 c	94.83±6.31 ab	-	-
		NPK	7.21±0.09 b	31.31±0.84 a	1.84±0.08 b	0.67±0.03 b	13.42±0.47 a	215.39±14.85 a	15.08±1.67 b	89.67±6.35 b	-	-
		NPKM	7.16±0.17 b	33.29±1.76 a	2±0.07 a	0.86±0.02 a	13.42±0.55 a	231.01±16.74 a	22.43±1.33 a	100.83±6.15 a	-	-
		NPKS	7.13±0.2 b	32.74±0.99 a	1.93±0.08 ab	0.7±0.07 b	13.05±0.54 a	216.42±8.24 a	13.72±1.40 b	99.33±7.74 ab	-	-
2016	Tillering stage	CK	7.52±0.14 a	30.11±1.15 b	1.87±0.11 a	0.63±0.02 c	15.99±0.24 a	134.74±0.84 b	5.95±3.54 c	86.92±6.29 b	6.67±4.12 b	0.77±0.37 ab
		NPK	6.56±0.14 c	32.77±3.32 ab	2.05±0.1 a	0.87±0.07 b	16.56±0.5 a	154.98±7.93 a	22.84±9.28 b	84.33±7.8 b	17.98±8.84 a	0.48±0.09 b
		NPKM	6.96±0.21 b	37.33±1.84 a	2.1±0.13 a	1.11±0.08 a	16.44±0.56 a	169.86±13.06 a	51.06±6.87 a	115.67±6.16 a	16.91±1.94 ab	1.13±0.27 a
		NPKS	6.62±0.15 c	34.9±2.71 ab	1.94±0.16 a	0.83±0.06 b	16.6±0.39 a	169.16±5.39 a	23.92±1.17 b	102.58±4.36 a	14.5±2.03 ab	0.5±0.03 b
	Heading stage	CK	7.61±0.21 a	30.34±1.03 b	1.76±0.06 b	0.69±0.05 c	17.26±0.95 a	151.31±4.52 a	6.31±4.27 b	68.75±6.94 a	7.22±1.19 a	0.6±0.07 a
		NPK	6.76±0.23 bc	30.91±1.67 b	1.78±0.09 b	0.83±0.06 bc	17.09±0.44 a	142.32±11.21 a	15.58±10.75 ab	59.33±17.73 a	8.08±3.36 a	0.6±0.27 a
		NPKM	7.2±0.25 ab	37.41±2.38 a	2.11±0.09 a	1.13±0.09 a	16.8±0.69 a	161.75±14.82 a	34.7±12.42 a	81.42±14.95 a	17.74±10.93 a	1.05±0.28 a
		NPKS	6.54±0.17 c	34.94±1.74 a	1.97±0.1 a	0.94±0.18 ab	17.04±0.59 a	173.92±28.32 a	20.14±6.87 ab	74.5±16.69 a	14.23±3.35 a	0.65±0.21 a
	Mature stage	CK	7.26±0.05 a	30.14±1.27 c	1.74±0.04 c	0.85±0.08 c	18.27±0.64 a	136.91±1.31 c	4.44±2.93 b	60.58±4 ab	13.66±4.68 a	4.94±2.96 b
		NPK	6.6±0.22 b	32.52±0.71 bc	1.9±0.04 b	0.97±0.09 bc	17.69±0.35 a	150.79±9.92 bc	9.89±4.02 b	48.33±5.64 b	8.41±0.37 a	8.21±3.56 ab
		NPKM	7.04±0.21 a	38.55±2.25 a	2.12±0.06 a	1.36±0.08 a	18±0.3 a	183.11±5.06 a	25.41±9.7 a	69.42±9.15 a	10.67±0.58 a	13.66±4.98 a
		NPKS	6.38±0.22 b	35.13±1.87 ab	2.02±0.1 ab	1.11±0.16 b	17.82±0.88 a	165.14±9.19 b	11.25±5.09 b	68.17±15.56 ab	9.54±2.02 a	11.71±1.76 ab

OM: Organic matter; TN, total N; TP: total P; TK: total K; AN: available N; AP: available P; AK: available K; NO_3-N: nitrate-nitrogen; NH_4-N: ammonium-nitrogen. CK: no fertilization; NPK: 100% chemical fertilizers; NPKM: 50% chemical fertilizers plus 6000 kg/ha pig manure; NPKS: 100% chemical fertilizers plus 8000 kg/ha crop straw. Different lowercase letters indicate significant differences between fertilization regimes ($P < 0.05$, Tukey's HSD test).

Table S2 The α-diversity indices of rhizosphere and endophytic diazotrophic communities during three growth periods in Jintan

	Growth period	Sample number	Richness (Chao1)	Evenness (Shannon)	PD	Coverage
Total rhizosphere diazotrophs in 2015	tillering stage	24	431.97±19.34 a	4.06±0.11 ab	9.84±0.39 a	0.997
	heading stage	24	422.84±21.64 a	3.9±0.1 ab	9.56±0.3 a	0.997
	mature stage	24	407.08±20.3 a	4.04±0.1 ab	9.32±0.19 a	0.997
Total endophytic diazotrophs in 2015	tillering stage	24	121.35±28.47 f	1.69±0.44 h	5.31±1 e	0.999
	heading stage	24	178.88±35.99 de	2.59±0.3 fg	6.78±0.71 cd	0.999
	mature stage	24	222.25±30.63 bc	3.17±0.19 de	7.76±0.52 b	0.999
Total rhizosphere diazotrophs in 2016	tillering stage	16	426.62±32.52 a	4.26±0.2 a	9.76±0.38 a	0.997
	heading stage	16	417.62±20.32 a	4.1±0.11 ab	9.52±0.33 a	0.997
	mature stage	16	421.53±27.69 a	4.08±0.07 ab	9.4±0.31 a	0.997
Total endophytic diazotrophs in 2016	tillering stage	15	140.33±31.34 ef	2.35±0.47 g	5.89±0.83 e	0.999
	heading stage	16	244.93±60.91 b	3.02±0.36 e	7.79±0.75 b	0.998
	mature stage	16	222.24±37.59 bc	2.93±0.48 ef	7.47±0.68 bc	0.998
Active rhizosphere diazotrophs in 2016	tillering stage	16	187.46±39.15 cd	3.77±0.24 bc	6.78±0.74 cd	0.999
	heading stage	16	225.24±45.36 bc	3.82±0.2 b	7.54±0.79 bc	0.999
	mature stage	16	144.34±57.11 ef	3.43±0.45 cd	5.81±1.25 e	0.999
Active endophytic diazotrophs in 2016	heading stage	15	120.83±58.64 f	2.6±0.75 fg	5.35±1.77 e	0.999
	mature stage	16	145.16±26.57 ef	2.91±0.35 ef	5.94±0.67 de	0.999

Different lowercase letters indicate significant differences of the α-diversity indices among different growth periods ($P < 0.05$, Tukey's HSD test).

Table S3 ANOSIM tests for the differences in the community composition between rhizosphere and endophytic diazotrophs among three growth periods

	**Tillering stage**		**Heading stage**		**Mature stage**	
	R	*P*	R	*P*	R	*P*
Total rhizosphere and endophytic diazotrophs in 2015	0.959	0.001	1	0.001	1	0.001
Total rhizosphere and endophytic diazotrophs in 2016	1	0.001	1	0.001	1	0.001
Active rhizosphere and endophytic diazotrophs in 2016	-	-	0.978	0.001	0.996	0.001

The ANOSIM analysis was run at the OTU level using the Bray-Curtis algorithm (n = 999 replications).

Table S4 Effects of growth period, fertilization regime and year on the α-diversity indices of rhizosphere and endophytic diazotrophs

**Factor**	**Total rhizosphere diazotrophs**			**Total endophytic diazotrophs**			**Active rhizosphere diazotrophs**			**Active endophytic diazotrophs**		
	**Richness (Chao1)**	**Evenness (Shannon)**	**PD**	**Richness (Chao1)**	**Evenness (Shannon)**	**PD**	**Richness (Chao1)**	**Evenness (Shannon)**	**PD**	**Richness (Chao1)**	**Evenness (Shannon)**	**PD**
Growth period (Gr)	*0.08**	*0.18****	*0.26****	*0.51****	*0.51****	*0.53****	*0.35****	*0.25****	*0.37****	*0.09***	*0.08**	*0.07**
Fertilization (Fe)	0.05	*0.06***	0.02	0.02	*0.04***	0.01	0.07	*0.1**	*0.08**	*0.25***	0.17	*0.25***
Year (Ye)	0	*0.22****	0	*0.05****	*0.05****	*0.03***	-	-	-	-	-	-
Gr × Fe	0.05	0.04	0.03	0.02	*0.05***	*0.05**	*0.23***	*0.25***	*0.25****	*0.26***	*0.25**	*0.33***
Gr × Ye	0.04	*0.05***	0.01	*0.04***	*0.09****	*0.04****	-	-	-	-	-	-
Fe × Ye	0.02	0	0.01	0	0.01	0.02	-	-	-	-	-	-
Gr ×Fe ×Ye	0.03	0.03	0.07	0.01	*0.03**	0.03	-	-	-	-	-	-

*Significance level: P < 0.05, *; P < 0.01, **; P < 0.001, ***. Significant differences (P < 0.05) are indicated in italics.*

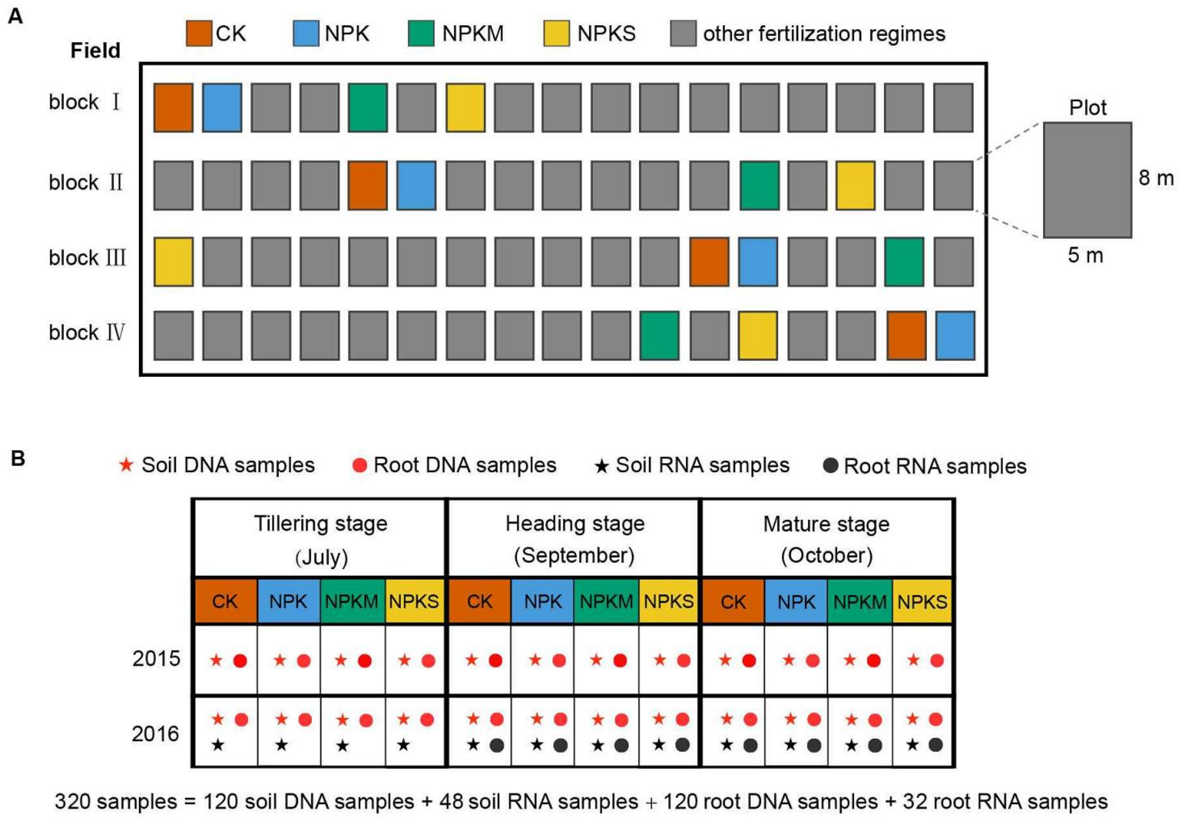

Figure S1 Field experimental design. **(A)** Field layout of the 68 plots (8 × 5 m^2 each) in a random block design of seventeen fertilization regimes with four replicates. The four fertilization regimes chosen in this study were as follows: no fertilization (CK), 100% chemical fertilizers (NPK), 50% chemical fertilizers plus 6000 kg/ha pig manure (NPKM) and 100% chemical fertilizers plus 8000 kg/ha crop straw (NPKS). **(B)** Sampling strategy. Rhizosphere soil samples and rice root samples were collected at the tillering, heading and mature stages of rice. In 2015, rhizosphere soil and root samples were collected for DNA extraction. In 2016, the rhizosphere soil and root samples were collected for DNA and RNA extraction, except the root samples collected at the tillering stage, which were only used for DNA extraction. In total, 120 rhizosphere soil DNA samples, 48 rhizosphere soil RNA samples, 120 root DNA samples and 32 root RNA samples were collected for subsequent *nifH* gene sequencing.

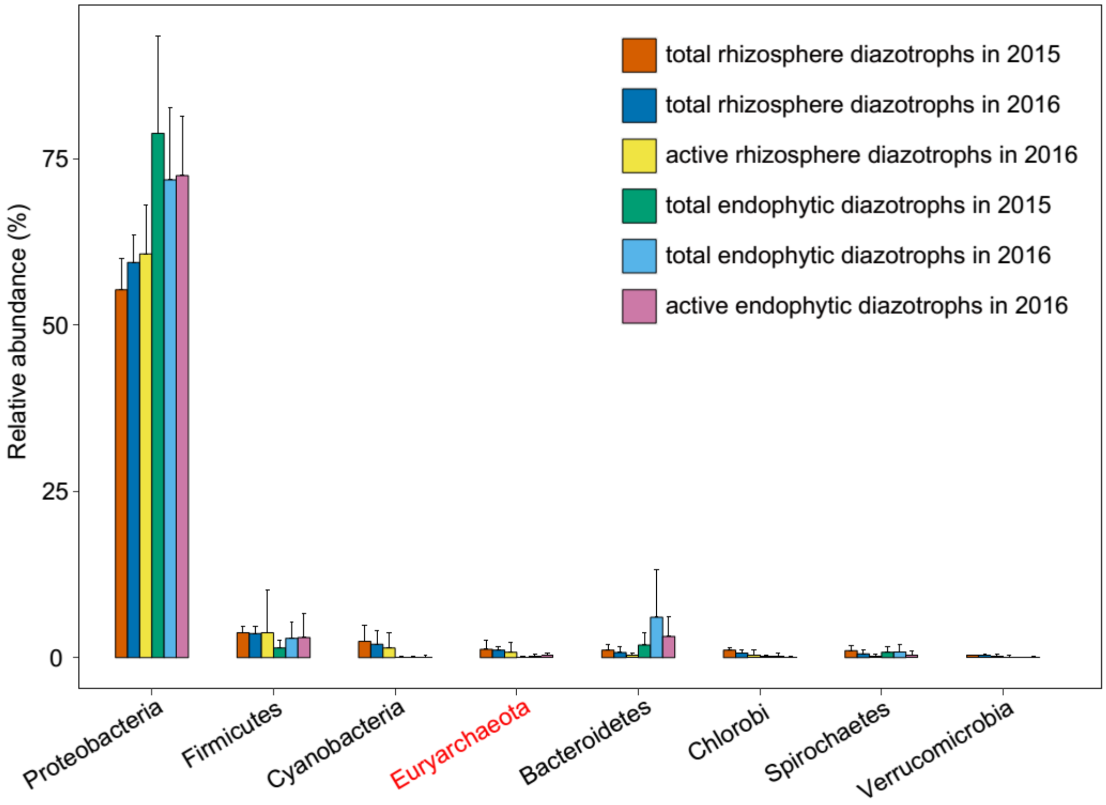

Figure S2 Bar plot showing the mean and standard deviation of the relative abundances of diazotrophic taxa at the phylum level across different sample types in 2015 and 2016. Euryarchaeota marked in red is one of the four phyla of the domain Archaea, and the other phyla belong to the domain bacteria.

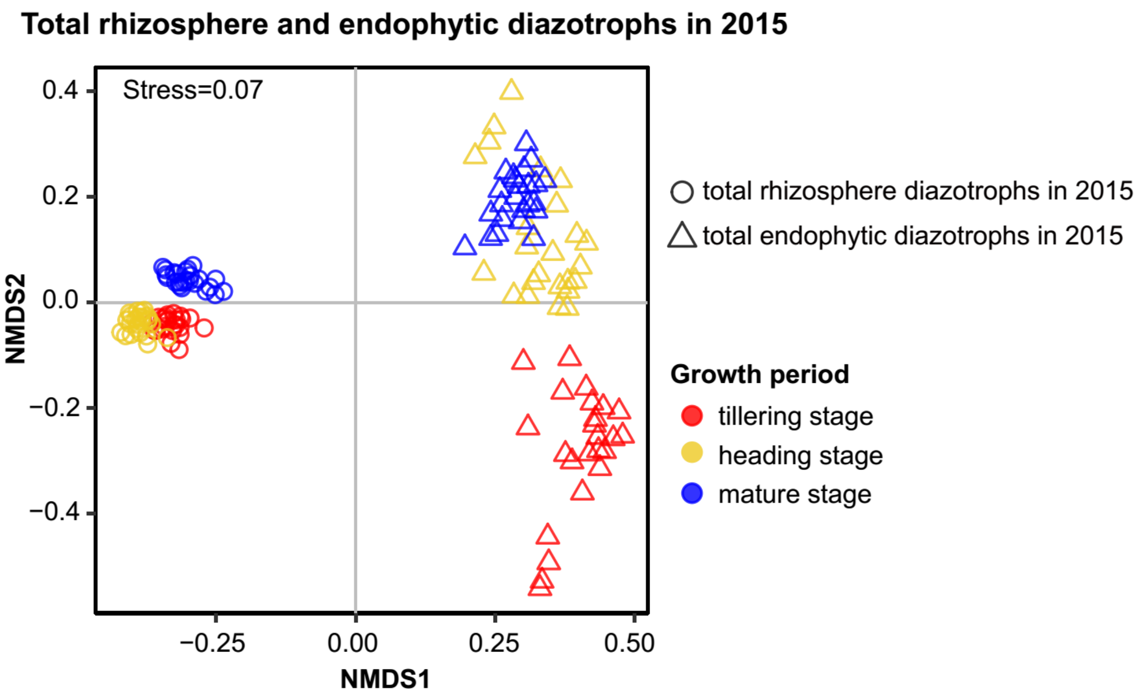

Figure S3 Effects of microhabitat and growth period on the community composition of total rhizosphere and endophytic diazotrophs in 2015. NMDS ordinations based on Bray–Curtis distance matrix of the total diazotrophic communities in rhizosphere soil and root samples.

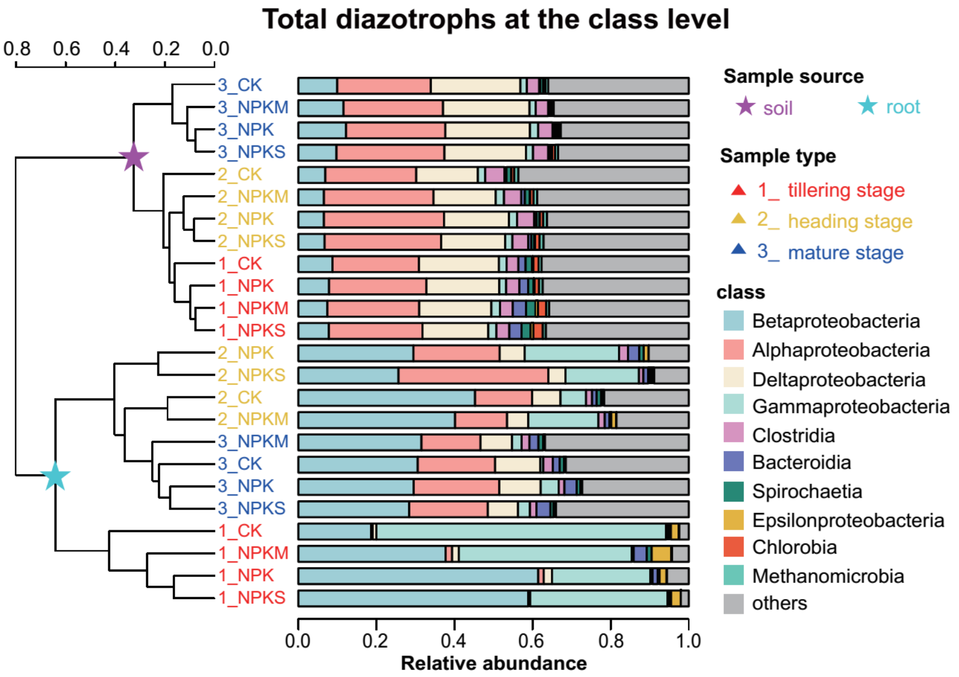

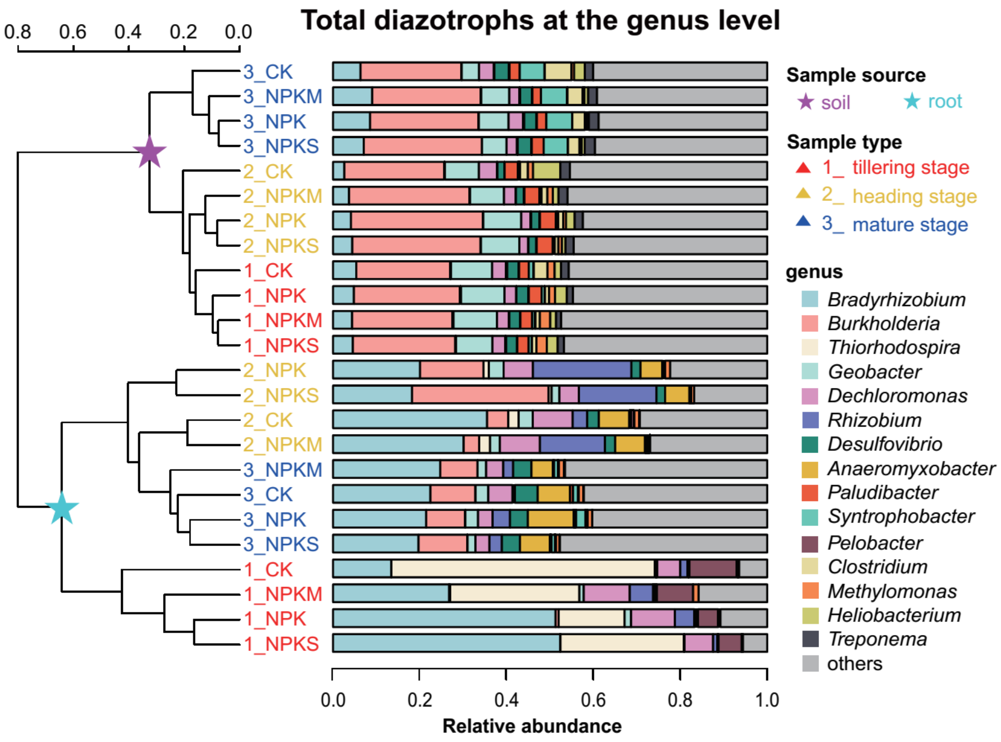

Figure S4 Community composition of total diazotrophs in rhizosphere soil and root samples at the class and genus levels in 2015. The UPGMA dendrogram was constructed based on Jaccard's similarity coefficient calculated from the OTU table.

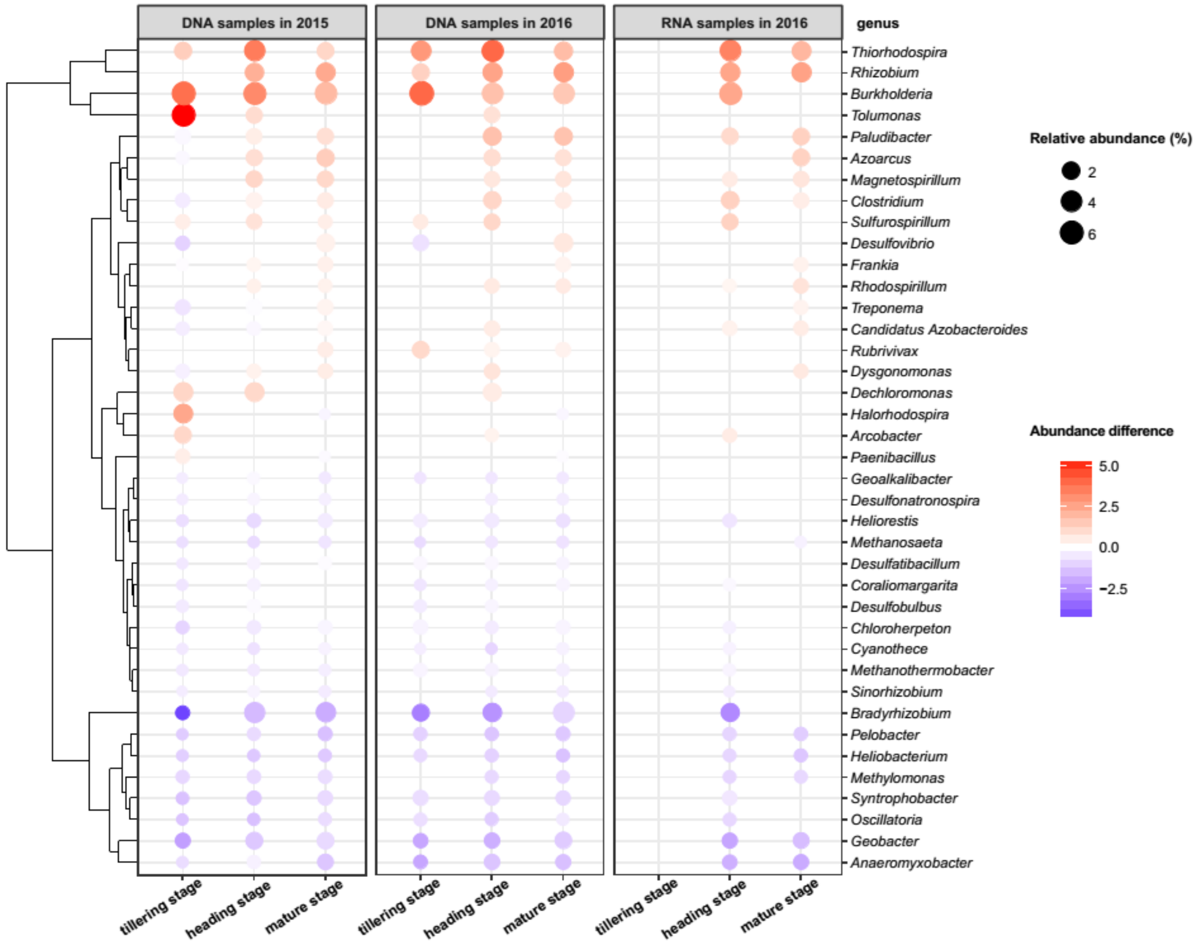

Figure S5 A bubble plot showing changes in the relative abundances of the dominant genera (relative abundance of diazotrophs > 0.1%) in the rhizosphere soil and root samples. The size of each bubble represents the square root transformed relative abundance percentage of each genus in the root samples, and the color represents the change in the relative abundance of each genus in the root and rhizosphere soil samples. Abundance difference = square root of the relative abundance percentage of a genus in the root sample - square root of the relative abundance percentage of the genus in the corresponding rhizosphere soil sample. Red bubbles indicate genera whose relative abundances are significantly higher in roots than in rhizosphere soil (*P* < 0.05, abundance change > 0), and blue bubbles indicate genera whose relative abundances are significantly lower in roots than in rhizosphere soil (*P* < 0.05, abundance change < 0). Cluster analysis of the genera was performed based on Euclidean dissimilarities using the complete linkage method.

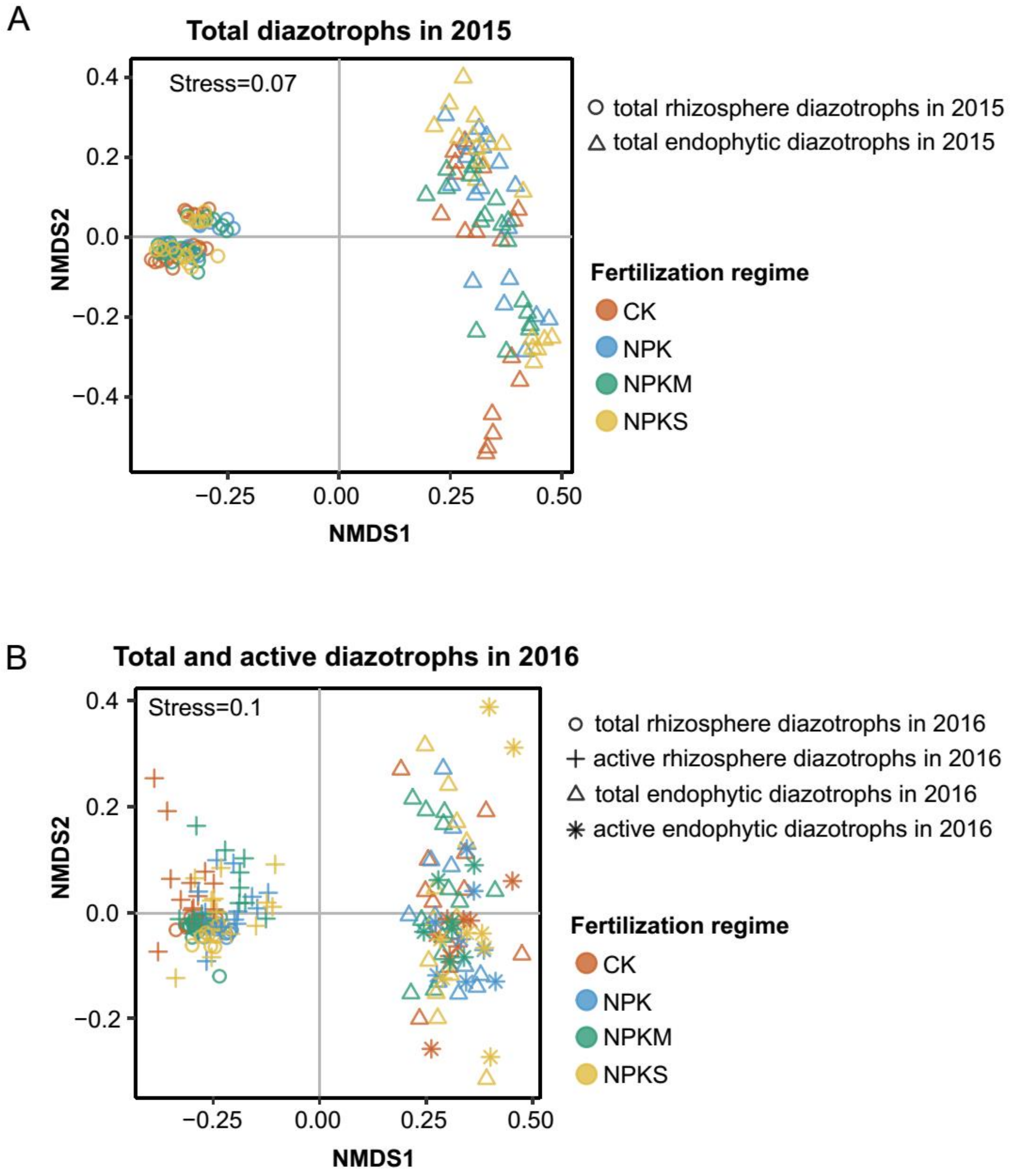

Figure S6 Effects of microhabitat and fertilization regime on the community composition of total and active root-associated diazotrophs in 2015 (A) and 2016 (B). NMDS ordinations based on Bray–Curtis distance matrix of diazotrophic communities among different fertilization regimes.

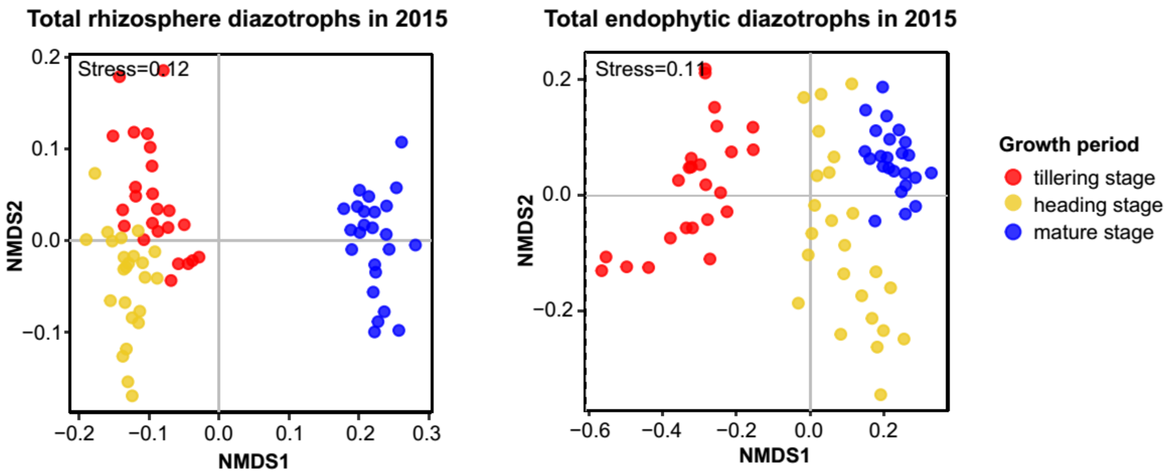

Figure S7 Effects of growth period on the community composition of total rhizosphere and endophytic diazotrophs in 2015. NMDS ordinations based on Bray–Curtis distance matrix of total diazotrophic communities in rhizosphere soil and root samples.

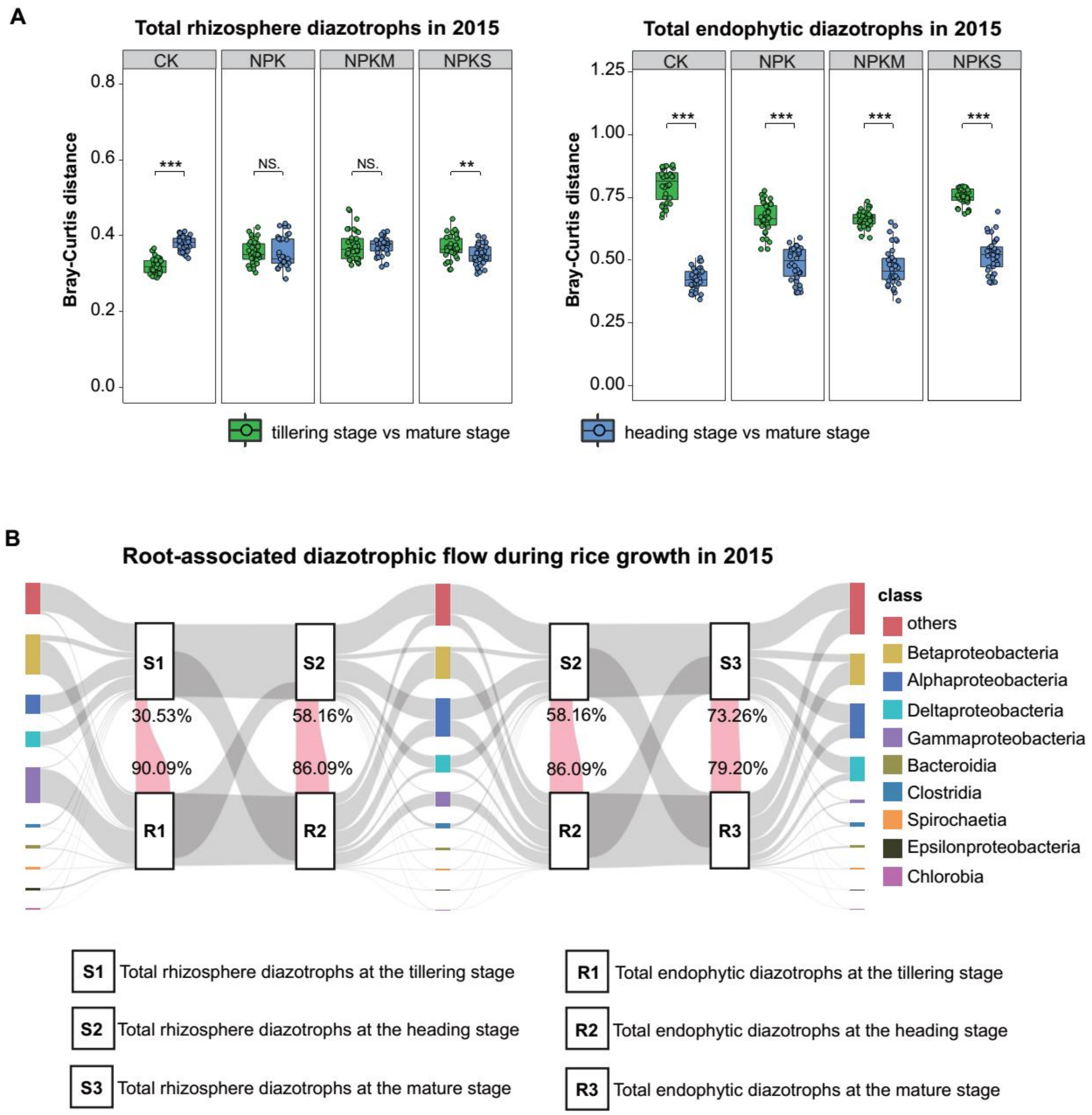

Figure S8 Temporal dynamics of total diazotrophic community composition in rhizosphere soil and root samples in 2015. **(A)** Bray–Curtis distances between the diazotrophic communities of soil DNA samples collected at the mature stage and samples collected at the other growth periods in 2015; the same analyses were performed in corresponding root DNA sample in 2015. Significance level: $P \geq 0.05$, ns; $P < 0.05$, *; $P < 0.01$, **; $P < 0.001$, *** (Student's *t*-test or Wilcoxon's rank-sum test). **(B)** Root-associated diazotrophic flows during rice growth at the class level in 2015. The far left, middle and far right bars indicate the community composition of rhizosphere and endophytic diazotrophs at the tillering, heading and mature stages, respectively. Different color bars indicate different classes, and the heights of these bars indicate the relative abundances of the classes. Each class and each sample source (total rhizosphere diazotrophs or total endophytic diazotrophs) at the same sampling point were

connected using gray bands as background. Pink flows represent variations in the relative abundance of OTUs shared by soil and root DNA samples at each sampling point. The numbers at the top of the pink flows indicate the percentages of the sequence numbers of these shared OTUs in the total sequence numbers of rhizosphere diazotrophs. The numbers at the bottom of the pink flows indicate the percentages of the sequence numbers of these shared OTUs in the total sequence numbers of endophytic diazotrophs.

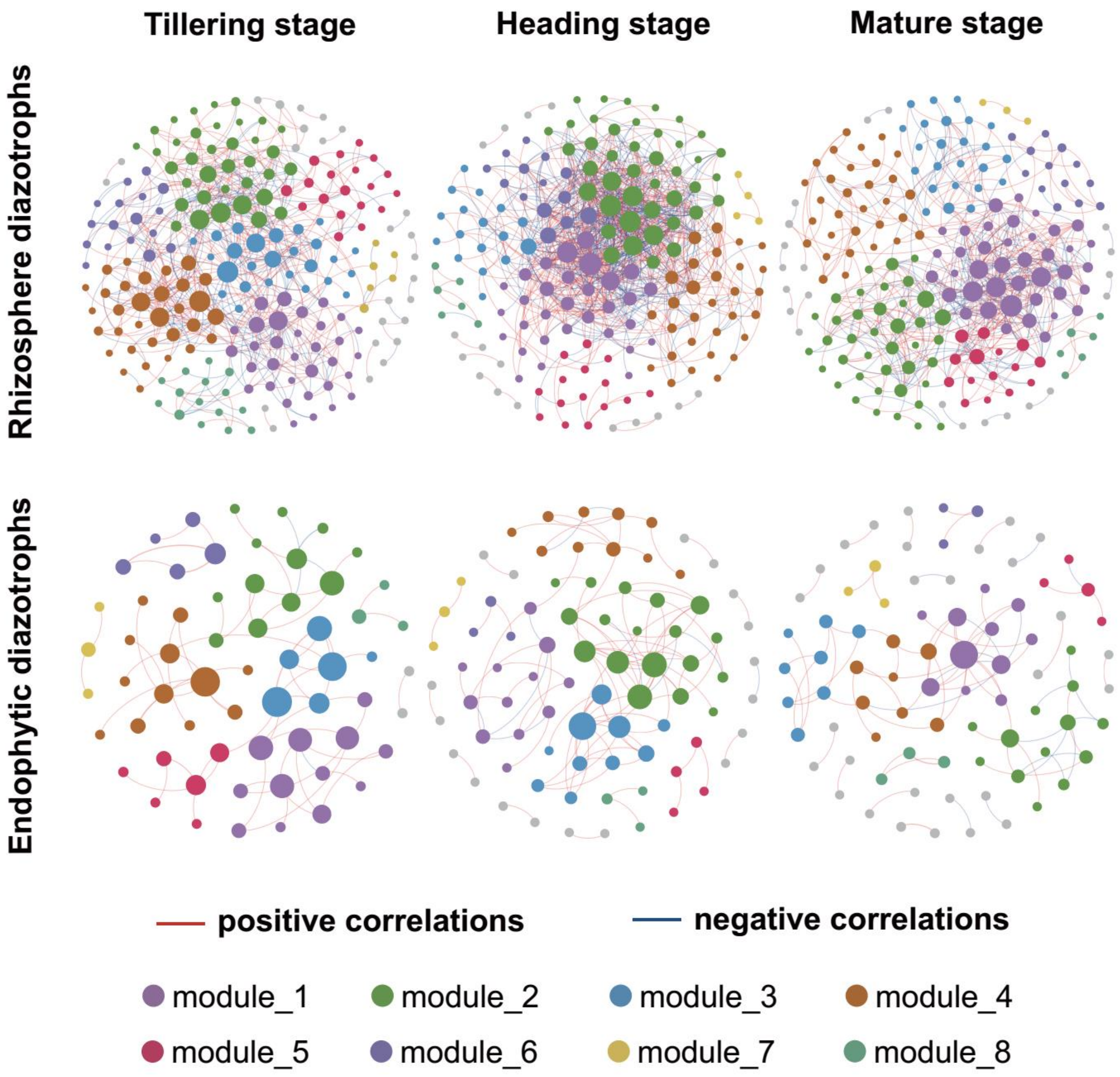

Figure S9 Co-occurrence networks of total rhizosphere and endophytic diazotrophic communities at three growth periods in 2015. Different nodes represent different OTUs. Edges represent Spearman's correlation relationships. Red solid lines show strong (Spearman's correlation coefficient r ＞ 0.65) and significant (P < 0.01) positive correlations between the nodes. Blue solid lines show strong (Spearman's correlation coefficient r < -0.65) and significant (P < 0.01) negative correlations between the nodes. The size of each point represents the node's weighted degree. Nodes in the eight largest modules are marked with different colors, while other nodes are marked with gray.
